# TREM-1 Interacts with Rotavirus Proteins and Drives Inflammatory Responses: A Combined Experimental and Computational Approach

**DOI:** 10.3390/pathogens14101029

**Published:** 2025-10-10

**Authors:** Amanda de Oliveira Matos, José Rodrigues do Carmo Neto, Fernanda Craveiro Franco, Jefferson do Carmo Dietz, Pedro Henrique dos Santos Dantas, Andrei Giacchetto Felice, Adriana Luchs, Milton Adriano Pelli de Oliveira, Artur Christian Garcia da Silva, Siomar de Castro Soares, Simone Gonçalves da Fonseca, Fátima Ribeiro-Dias, Bruno Junior Neves, Carolina Horta Andrade, Marcelle Silva-Sales, Helioswilton Sales-Campos

**Affiliations:** 1 Instituto de Patologia Tropical e Saúde Pública, Universidade Federal de Goiás, Goiânia 746050-050, GO, Brazil; amandamatos@ufg.br (A.d.O.M.); rodriguesnneto@gmail.com (J.R.d.C.N.); fec_franco@ufg.br (F.C.F.); pedrohenrique1997@discente.ufg.br (P.H.d.S.D.); mapoliv@ufg.br (M.A.P.d.O.); sfonseca@ufg.br (S.G.d.F.); fdias@ufg.br (F.R.-D.); 2 Instituto de Ciências Biológicas e Naturais, Universidade Federal do Triângulo Mineiro, Uberaba 38025-180, MG, Brazil; andreigf@hotmail.com (A.G.F.); siomars@gmail.com (S.d.C.S.); 3 Instituto Adolfo Lutz, São Paulo 01246-000, SP, Brazil; driluchs@gmail.com; 4 Faculdade de Farmácia, Universidade Federal de Goiás, Goiânia 74605-170, GO, Brazil; artur_silva@ufg.br (A.C.G.d.S.); brunoneves@ufg.br (B.J.N.); carolina@ufg.br (C.H.A.)

**Keywords:** rotavirus, diarrheal diseases, acute gastroenteritis, TREM1, immune response

## Abstract

Rotavirus (RV) is one of the main etiologic agents associated with diarrheal diseases (DDs), being responsible for approximately 200 thousand deaths annually. Currently, there are still many aspects regarding the virus biology, cell cycle, and pathophysiology of RV that need further elucidation. Therefore, the present work aimed to investigate whether the triggering receptor expressed on myeloid cells 1 (TREM-1) might be associated with RV infection. This immune receptor has been observed as an amplifier of inflammatory responses in different infectious and non-infectious diseases, including inflammatory bowel disease and celiac disease. Initially, we searched for public transcriptomic data regarding RV infection and the expression of TREM-1 and its associated genes, which were significantly upregulated in infected mice and children. Then, we infected monocytes with the virus, with or without a TREM-1 inhibitor. The inhibition of the receptor’s activity resulted in a significant decrease in IL-1β production. We also observed a reduction in cytopathic effects when MA104 cells were treated with TREM-1 inhibitors and then infected with simian RV. To further elucidate the interactions between the virus and TREM-1, in silico tools were used to simulate interactions between the receptor and RV proteins. These simulations suggested the occurrence of interactions between TREM-1 and VP5*, a protein involved in viral attachment to target cells, and also between the receptor and NSP4, a viral enterotoxin with immunostimulant properties. Hence, our results indicate that TREM-1 is involved in RV infection, both as a mediator of inflammatory responses and as a player in the host–virus relationship.

## 1. Introduction

Diarrheal diseases (DD) are a major public health concern worldwide, causing over 1 million deaths in 2021, especially in low- and middle-income countries [1,2]. Characterized by frequent loose or liquid stools, usually accompanied by abdominal pain, vomiting, and fever, this condition is caused by several pathogens, including viruses from the *Rotavirus* (RV) genus [3]. Although vaccination has drastically decreased the number of deaths and hospitalizations associated with RV [4,5], this pathogen is still considered a pathogen of concern: in 2021, it was considered the main cause of diarrheal deaths across all ages, predominantly in children younger than 5 years, accounting for approximately 176,000 (131,000–230,000) deaths globally [1].

RV is a non-enveloped, double-stranded RNA virus belonging to the *Sedoreoviridae* family. Currently, there are nine RV species accepted by the International Committee on Taxonomy of Viruses (ICTV), with *Rotavirus alphagastroenteritidis* (RVA) accounting for over 95% of the infections in humans and animals [6,7,8]. Structurally, the virus is composed of a icosahedral triple-layered capsid formed by six structural proteins: VP7 and VP4 form the outer layer and VP6, the middle layer, while VP2 forms the viral core, anchoring the segmented viral genome and the VP1 and VP3 proteins, which are associated with viral replication [7,9,10,11,12,13]. Besides the triple-layered particle (TLP), RV can also be found as a double-layered particle (DLP), which is formed after the internalization of the TLPs, when the VP7/VP4 layer is lost in phagosomes [7].

RV transmission mainly occurs through the fecal–oral route and targets mature enterocytes from the intestinal villi as its main replication target [3]. During the infectious process, the VP4 protein, which constitutes the viral spike, is proteolytically cleaved into two domains: VP8*, which recognizes glucans on the cells surface, and VP5*, which acts posteriorly to VP8* and interacts with proteins, such as integrins and the 70 kilodalton heat shock protein (Hsp70) [14,15,16,17,18,19,20,21,22,23]. RV infection leads to serious damage of the intestinal tissues, resulting in profuse aqueous diarrhea [7,24]. Nonetheless, the systemic nature of the RV infection has recently been explored, and it has been associated with the occurrence of seizures, encephalitis, pneumonia, and myocarditis, among other symptoms [25]. Another important feature of the infection is the activity of the non-structural protein NSP4, which can be found in a secreted form and act as an enterotoxin, being able to trigger diarrhea [26]. Currently, RV-induced diarrhea is mainly treated by rehydration, probiotics, and treatment of symptoms, with no specific antiviral being licensed against it yet. Nonetheless, many candidate drugs and natural compounds have been tested, and the antiparasitic drug nitazoxanide has been suggested as effective in the control of the virus [27,28,29,30,31].

So far, the innate immune response to RV has been mainly associated with the intracellular receptors RIG-I (retinoic acid-inducible gene I protein) and MDA-5 (melanoma differentiation-associated protein 5) [32]. These receptors detect the viral genome and signalize through MAVS (mitochondrial antiviral-signaling protein) for the expression of type I and III interferons (IFN) and IFN-stimulated genes (ISGs), which will induce apoptosis of infected cells and the antiviral state [33,34,35,36]. In addition, other immune receptors such as TLR3 (toll-like receptor 3), NLRP9 (NACHT, LRR, and PYD domain-containing protein 9) and NLRC4 (NLR family CARD domain-containing protein 4), have also been implicated in the immune response against RV, but these processes are still elusive [37,38]. 

Among the immune receptors, the triggering receptor expressed on myeloid cells-1 (TREM-1) has been increasingly explored in infectious diseases, including viral infections, due to its role in the amplification of inflammatory responses, and is now considered as a promising therapeutic target and diagnostic/prognostic biomarker in different scenarios [39,40,41]. Although mainly explored in bacterial infections, TREM-1 signaling has been implicated in exacerbated immune responses to viruses, such as HIV, influenza, enterovirus A71, West Nile fever, and Marburg virus [42,43,44,45,46,47,48]. Therefore, the receptor seems to play a significant, yet underexplored, role in the immunopathology of several viral diseases.

TREM-1 can be observed on the surface of neutrophils, monocytes, dendritic cells, besides epithelial and endothelial cells of different organs, including the gastrointestinal tract [49,50,51,52,53,54,55]. Following its activation, the receptor signals through the adaptor protein, DAP12, leading to the expression of inflammatory cytokines and chemokines (including TNF-α, IL-1β, IL-6, IL-8, CCL2, CCL3 and CCL7), the release of NETs and reactive oxygen species (ROS) from neutrophils, among other features [50,56,57,58,59,60,61,62]. The recognition and activation processes of TREM-1 still demand further clarification, including the nature of its ligands and the elucidation of how they interact with each other. So far, only four ligands have been confirmed: extracellular actin and extracellular cold-inducible mRNA binding protein (eCIRP), the peptidoglycan recognition protein 1 (PGLYRP1) and high mobility group protein B1 (HMGB1) [63,64,65,66], although others, from infectious origins, have been proposed. In this scenario, a putative interaction between TREM-1 and RV has never been explored before. Therefore, we combined in vitro assays and bioinformatics tools to investigate whether TREM-1 is involved in host–virus interactions with RV and what is the impact of its activation for the infection outcome.

## 2. Materials and Methods

### 2.1. Analysis of Public Transcriptomic Data

Data regarding gene expression during RV infection was obtained from the public database Gene Expression Omnibus (GEO, https://www.ncbi.nlm.nih.gov/geo/ (accessed on 15 September 2025)—National Institutes of Health, Bethesda, MD, USA). The datasets were selected based on the following criteria: availability of expression values, a minimum number of three samples/individuals (N) per group, the inclusion of the TREM-1 gene (formal nomenclature: *TREM1* for the human gene and *Trem1* for the murine gene) in the searched genes and the availability of the GEO2R tool within the dataset. Next, the groups were selected, and the differentially expressed genes (DEGs) were defined using the Benjamini–Hochberg method. The list of DEGs was retrieved and used to generate Volcano plots (log2 fold change [log2FC] cutoff of 1 and *p* < 0.05) in the SRPLOT server (https://www.bioinformatics.com.cn/srplot (accessed on 15 September 2025)—Changsha, Hunan, China) [67]. The expression values of TREM-1 and the genes associated with its signaling pathway were searched (based on the QIAGEN’s TREM-1 signaling gene list, https://geneglobe.qiagen.com/us/knowledge/pathways/trem1-signaling (accessed on 15 September 2025)—QIAGEN, Germantown, MD, USA) and used for subsequent analysis. Principal components analysis (PCA) of the TREM-1 pathway during RV infection was performed in SRPLOT and, for the generation of heatmaps, the expression values were submitted to a min–max normalization, and the maps were created with the GraphPad Prism version 9.0 (GraphPad Prism Software, Boston, MA, USA).

### 2.2. Cell and Virus Cultivation

MA104 cells (simian renal epithelial lineage) were acquired from the Instituto Adolfo Lutz cell bank (São Paulo, SP, Brazil) while THP-1 cells (human monocyte cell lineage) were kindly provided by Dr. Artur Christian Garcia da Silva (Laboratório de Ensino e Pesquisa em Toxicologia In Vitro, Universidade Federal de Goiás, Goiânia, GO, Brazil). MA104 cells were cultivated in Dulbecco’s Modified Eagle’s Medium with high glucose (DMEM-HG) (D6429, Sigma-Aldrich, St. Louis, MO, USA), supplemented with 10% fetal bovine serum (F7524, Sigma-Aldrich, St. Louis, MO, USA) and 1% penicillin-streptomycin (10.000 units/mL of penicillin and 10,000 µg/mL of streptomycin, P0781, Sigma-Aldrich, St. Louis, MO, USA). Subculture was performed when a 90% confluence was reached, using trypsin-EDTA (2.5 g/mL, Vitrocell, Campinas, SP, Brazil) to detach the cells from the culture flasks. THP-1 cells were cultivated in RPMI Medium 1640 (31800022, Gibco, Grand Island, NY, USA, containing 1.5 g/L of sodium bicarbonate and 1 mM of sodium pyruvate), supplemented with 2 mM of L-glutamine (200 mM, G7513, Sigma-Aldrich, St. Louis, MO, USA), 1% penicillin-streptomycin (10,000 units/mL of penicillin and 10,000 µg/mL of streptomycin, P0781, Sigma-Aldrich, St. Louis, MO, USA), and 10% fetal bovine serum (F7524, Sigma-Aldrich, St. Louis, MO, USA). The cells were maintained in a CO_2_ incubator at 37 °C and 5% CO_2_.

The RRV (rhesus rotavirus, passage #13) strain of RV was provided by Dr. Adriana Luchs (Instituto Adolfo Lutz) and was expanded in MA104 cells. In this regard, RV was activated (with trypsin at 10 µg/mL for 30 min at 37 °C) and added to the MA104 cell monolayer in 75 cm^2^ flasks, at a multiplicity of infection (MOI) of 0.1, in incomplete medium (DMEM-HG with 1% penicillin-streptomycin). After the complete lysis of the cells (approximately 72 h of infection followed by 3 freezing/thawing cycles), the cell lysate was centrifuged (2000 rpm, 25 °C for 10 min) and filtered (Millipore Syringe Filter, 0.22 µm—EMD Millipore Corporation, Burlington, MS, USA) to remove debris, and the aliquots were stored in a −20 °C freezer until use.

### 2.3. Viral Titration

To quantify the viral load, the 50% tissue culture infectious dose (TCID50) was calculated. In this process, MA104 cells were plated in 96-well plates (4 × 10^4^ cells/well) and infected with 10 different dilutions of the viral stock (10^−1^ to 10^−10^) in incomplete medium supplemented with 0.5 μg/mL of trypsin-EDTA. After 5 days in the CO_2_ incubator, the cells were analyzed under an optical microscope to observe cytopathic effects due to RV infection, and the number of wells with cytopathic effect per dilution was used to calculate the TCID50, using the Reed–Muench method [68]. Later, we converted the TCID50 value to PFU (plaque-forming units), using the following formula PFU = TCID50 × 0.7 [69].

### 2.4. Cytokine Production in THP-1 Cells

To assess the production of pro-inflammatory cytokines, we used undifferentiated THP-1 cells either treated or not treated with the TREM-1 inhibitor VJDT (HY-157122, 98.96% purity, MedChemExpress, Monmouth Junction, NJ, USA), either in the presence or not in the presence of RV infection. In this process, 1 × 10^5^ cells/well were plated in 48-well plates with complete RPMI medium, either supplemented or not supplemented with 25 µM of the TREM-1 inhibitor, VJDT. After one hour of incubation with VJDT, RV was added to the cells at a MOI of 5, followed by a 24-h incubation period. Lipopolysaccharide (LPS, L4516, Sigma-Aldrich, St. Louis, MO, USA) at 1 µg/mL was used as a positive control. The plates were then centrifuged (1500 rpm, 7 min, 25 °C) and the supernatants were collected and stored in an −80 °C freezer until use. All assays were conducted in two independent experiments with four replicates per group.

### 2.5. Cytokine Production

The levels of TNF-α and IL-1β were quantified in the cell culture supernatants by enzyme-linked immunosorbent assays (ELISA), using BD OptEIA™ kits (TNF-α: 555212, IL-1β: 557953, BD Biosciences, San Diego, CA, USA) according to the manufacturer’s instructions. The plates were read by an Agilent BioTek 800 TS microplate reader (Agilent Technologies, Inc., Santa Clara, CA, USA) at 450 nm, with a correction at 570 nm.

### 2.6. Analysis of Cytopathic Effects

To observe whether TREM-1 inhibition impacted RV infection, we used two different inhibitors: VJDT and the LR12 peptide (LQEEDAGEYGCM, 95.84% purity, purchased from Biomatik, Kitchener, ON, Canada). For these experiments, MA104 cells were cultivated in 96-well plates (4 × 10^4^ cells/well) for 24 h and treated with the inhibitors for 1 h (LR12 at concentrations of 25, 50, and 100 μg/mL, and VJDT at 12.5, 25, and 50 μM, in incomplete DMEM-HG medium). Later, activated RV diluted in incomplete medium was added to the cells at MOI 0.1 and was incubated for 1 h to allow viral adhesion.

For the LR12 experiments, the activated viral stock was treated for 30 min with a protease inhibitor cocktail (P8340, Sigma-Aldrich, St. Louis, MO, USA) to inhibit the trypsin used to activate RV. The cocktail was added in volumes that resulted in 1 mM of the AEBSF (4-(2-aminoethyl)benzenesulfonyl fluoride) inhibitor in the final viral solution. After the 1-h incubation, the supernatants were removed, and fresh incomplete medium containing 0.5 μg/mL of trypsin was added to the cells. For the experiments with VJDT, the TREM-1 inhibitor was also included in the fresh incomplete medium, with 0.5 µg/mL of trypsin added after 1 h of incubation, at the same concentrations previously described (12.5, 25, and 50 μM). 

For both experiments with LR12 and VJDT, the plates were maintained in a CO_2_ incubator and, after 5 days, the supernatant was collected, the cells were washed twice with PBS, and incomplete medium containing 10% of a 5 mg/mL MTT solution (3-(4,5-dimethylthiazol-2-yl)-2,5-diphenyl-2H-tetrazolium bromide, Exodo Científica, Sumaré, São Paulo, Brazil) was added to the wells. Following a 3-h incubation, the supernatant was collected, DMSO was added to solubilize Formazan crystals, and the plates were analyzed in a Agilent BioTek 800 TS microplate reader (Agilent Technologies, Inc., Santa Clara, CA, USA) at 570 nm wavelength.

### 2.7. Statistical Analysis

The GraphPad Prism 9.0 software (GraphPad Software, Boston, MA, USA) was used for the statistical analysis of transcriptomic and in vitro data. The Shapiro–Wilk test was used to verify the normality of the data, and the following tests were used to search for significant differences (*p* < 0.05) between the groups: for comparison of two groups we used Student’s *t*-test (parametric data) or Mann–Whitney’s test (non-parametric data), while for the comparison of 3 or more groups we used a one-way ANOVA (parametric data) or Kruskal–Wallis test (non-parametric data), with Turkey’s test and Dunn’s test as post hoc tests, respectively.

### 2.8. In Silico Analysis of Putative Interactions Between TREM-1 and RV Proteins

We searched on the RCS PDB website (https://www.rcsb.org/ (accessed on 15 September 2025)) [70] for PDB files corresponding to TREM-1, RVA TLP, and RVA DLP capsomeres, besides VP5* and NSP4 proteins from different RVA genotypes (P[4], P[6], P[8], P[9], E1, E2, and E3). The selected genotypes are representative of the three human RVA genogroups (Wa-like, DS-1-like, and AU-1-like), and are also the most epidemiologically prevalent genotypes of RVA. We also searched for the structures of VP5* and NSP4 proteins from the SA-11 and UK bovine strains, respectively, and the integrin alpha-4 (ITGA4) and TLR2 human proteins to act as positive controls [71,72]. Protein structures that could not be retrieved from the RCS PDB or the AlphaFold database were predicted, and the putative interactions between TREM-1 and RV were observed with molecular docking and molecular dynamics simulations.

### 2.9. Protein Modeling

The VP5* structures (from P[4], P[6], P[8], and P[9] genotypes and from the SA-11 strain) and NSP4 (from E1, E2, and E3 genotypes and from the UK bovine strain) were not found in PDB, so they were predicted with an artificial-intelligence-based method, AlphaFold2, via ColabFold [73], with amino acid sequences retrieved from NCBI’s Virus (https://www.ncbi.nlm.nih.gov/labs/virus/vssi/#/ (accessed on 15 September 2025)) and Genbank (https://www.ncbi.nlm.nih.gov/genbank/ (accessed on 15 September 2025)). For the modeling process, we chose to search for template models in the pdb100* database and to conduct post-prediction relaxation of the five generated models with the Amber force-field. The best ranked models were selected and analyzed regarding their structural parameters with the Molprobity webserver [74], which was also used to generate the Ramachandran plots. The structure of human HSPA8 was retrieved from the AlphaFold database (https://alphafold.ebi.ac.uk (accessed on 15 September 2025)), while the structure of TLR2 was obtained from RCS PDB.

### 2.10. Molecular Docking

Rigid molecular docking simulations were conducted with the Cluspro webserver [75,76,77]. The 10 best ranked poses in the “balanced” coefficient were then re-ranked according to the “lowest energy” score, and the one with the most negative value was selected for molecular dynamics (MD) simulations for each protein complex.

### 2.11. Molecular Dynamics (MD)

To assess the stability of the predicted complexes, MD simulations were conducted with the selected docked poses using the WebGRO webserver, which is based on GROMACs 2025.3 software (GROMACS, Groningen, The Netherlands) [78]. The simulations were conducted in triplicates, with the GROMOS 43a1 forcefield in an octahedron box type with a SPC water model neutralized with 0.15 M of NaCl. For the minimization parameters, a steepest descent integrator with 5000 steps was used. The NVT/NPT equilibration type was used and the simulation ran in 310 K, at 1 bar for 50 ns, with approximately 1000 frames per simulation. The root mean square deviation (RMSD) graphs were generated in the GraphPad Prism 9.0 software, with the output data from WebGro.

### 2.12. Interaction Interfaces and Energetic Parameters of Protein–Protein Complexes

The interaction interfaces of the protein–protein complexes were determined with the combined results of analysis with the PPCheck [79] and PDBsum Generate [80] webservers, in addition to the DIMPLOT software (LigPlot+ v.2.2 suite—https://www.ebi.ac.uk/thornton-srv/software/LigPlus/manual2/manual.html (accessed on 15 September 2025)) [81]. The energetic parameters of the complexes were analyzed with the HawkDock (binding free energy calculation through the MM/GBSA method) [82], Prodigy (dissociation constant [Kd] at 36.5 °C) [83], CCharPPI [84] (using the PYDOCK_TOT [total binding energy], FA_ATR [attractive van der Waals energy], DESOLV [desolvation energy], ELE [total electrostatic energy], VDW [van der Waals energy], HBOND [hydrogen bonding potential] descriptors), and PIZSA [85] (8 Å threshold) webservers.

## 3. Results

### 3.1. TREM-1 Pathway Is Upregulated in Mice and Children Infected with RV

Following our selection criteria, three studies were selected for the transcriptomics analysis: GSE41594 [86], GSE50628 [87] and GSE69529 [88]. GSE41594 was conducted with newborn BALB/c mice that were intraperitoneally (i.p.) infected with the rhesus monkey RVA strain (RRV) or inoculated with a saline solution (control animals). They were divided into six experimental groups with three animals each (control, CT, and infected, RV, in three time periods—3, 7, and 14 days post-infection [p.i.]), and gene expression was analyzed in samples from the gallbladder and extrahepatic bile ducts by microarray [86]. Both GSE69529 and GSE50628 were clinical studies conducted with children. The first, enrolled children (<10 years) with confirmed DD induced by RV (*n* = 55) and healthy controls (*n* = 31) [88]. The gene expression analysis was performed by RNAseq, with whole blood samples collected from one to eight days after disease onset [88]. GSE50628 included six children (<three years) with confirmed RV infection who were divided into two groups: with uncomplicated DD (RV CT) or DD with complex febrile seizures (RV SE) [87]. Whole blood samples were collected in the acute (AG—two and four days after symptom onset) and recovery phases (RE—seven to eleven days after symptom onset) of the disease, with the expression profiles determined by microarray [87].

**Figure 1:**
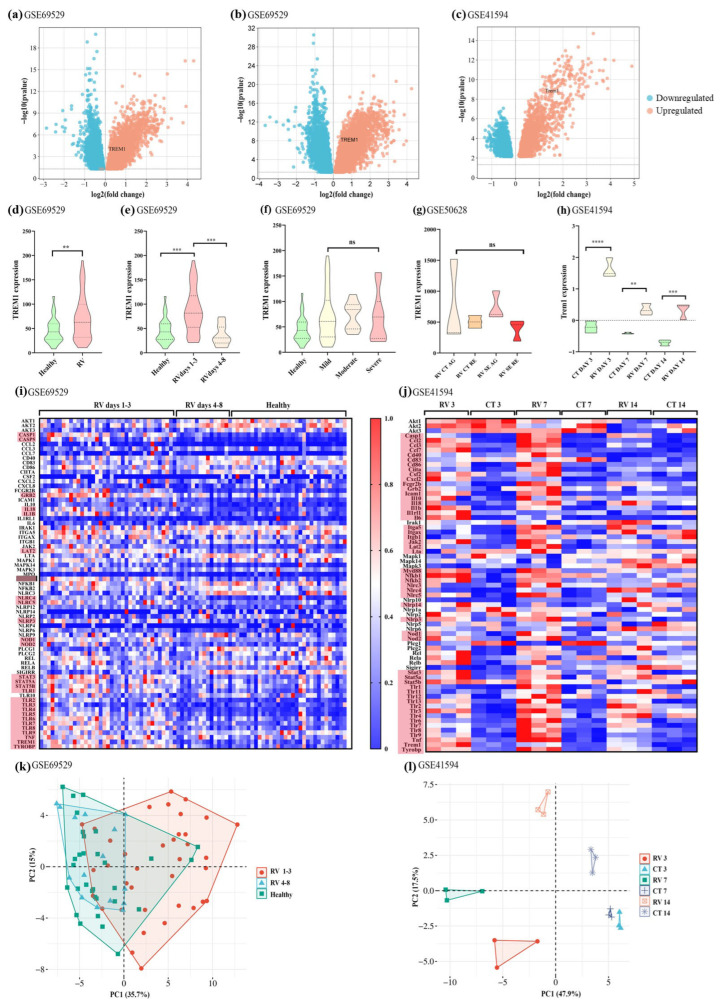
Analyses of publicly available transcriptomic data from children and mice infected with rotavirus (RV). Volcano plots comparing gene expression of children with RV and healthy controls from the GSE69529 dataset (**a**), children with RV infection on days 13 of disease and healthy controls from the GSE69529 dataset (**b**), and mice experimentally infected with RV on day 3 post-infection from the GSE41594 dataset (**c**). (**d**) Comparison of *TREM1* expression in healthy and infected children (GSE69529) with Mann–Whitney’s test. (**e**) Comparison of *TREM1* expression in children with RV on days 1–3 and 4–8 of disease onset and in healthy controls (GSE69529) with the Kruskal–Wallis test (Dunn’s test as a post hoc test). (**f**) Comparison of *TREM1* expression in healthy controls and in children with mild, moderate, and severe forms of RV-induced DD (GSE69529), with the Kruskal–Wallis test. (**g**) Comparison of *TREM1* expression in children with RV-induced DD, with (RV SE) or without seizures (RV CT), during the acute (AG) and recovery (RE) stages (GSE50628), with the Kruskal–Wallis test. (**h**) Comparison of *Trem1* expression in RV-infected or control mice after 3, 7, or 14 days post-infection (GSE41594) with one-way ANOVA (Turkey’s test as a post hoc test). **** *p* < 0.0001, *** *p* < 0.001, ** *p* < 0.01, ns = non-significant. Heatmaps showing the expression of genes associated with *TREM1* expression from the human dataset (GSE69529) (**i**) and murine dataset (GSE41594) (**j**). Genes with the most expressive difference between groups are highlighted in red. Principal component analysis (PCA) for the *TREM1* signaling pathway genes from the human dataset (GSE69529) (**k**) and murine dataset (GSE41594) (**l**).

In the GSE69529 dataset, *TREM1* was among the upregulated genes in infected children when compared to healthy controls (log2FC of 1.013, *p* = 0.00021) (Figure 1a). When the groups’ medians were compared by Mann–Whitney’s test, a significant difference between the healthy and infected groups (*p* = 0.0083) was observed (Figure 1d). The receptor’s expression also varied according to the day of infection (Kruskal–Wallis test *p* < 0.0001), with significant differences being observed between the healthy group and children with DD on days 1 to 3 of disease onset (Dunn’s test *p* < 0.0003), but not on days 4 to 8 (Figure 1e). The samples from days 4 to 8 had significantly lower values of *TREM1* expression (Dunn’s test *p* = 0.0001) compared to those from days 1 to 3 (Figure 1e) and were similar to the control group (Dunn’s test *p* > 0.999). Indeed, with only data obtained on days 1–3 of disease onset, compared to the control group, a log2FC of 1.367 (*p* = 0.000000023, Figure 1b) was observed in the Volcano plot, thus indicating a 2.6 increase in the expression of *TREM1* in infected individuals in comparison to healthy children.

Regarding disease severity, the expression of the receptor did not significantly vary with the comparison of mild, moderate, and severe cases from the GSE69529 study (Figure 1f). Similarly, analysis of the GSE50628 dataset did not indicate *TREM1* as an upregulated gene when comparing children with standard DD compared to those with DD accompanied by seizures, either during the acute or recovery phases (Figure 1g). 

Regarding the murine dataset (GSE41594), *Trem1* was among the upregulated genes in infected mice in comparison to their non-infected counterparts (control) after three days of infection (log2FC of 1.842, *p* = 2.53513E-09, Figure 1c). The expression values of *Trem1* were compared by one-way ANOVA between control and infected animals on each day of infection, with significant differences observed in the three different timepoints (Turkey’s test *p* < 0.0001, 0.0075, and 0.0005, for days 3, 7, and 14, respectively, Figure 1h). Nonetheless, a time-dependent decrease in the expression of the receptor was observed in the comparison of the RV group on days 3 and 7 post-infection (Turkey’s test *p* < 0.0001, Figure 1h), as was observed in the dataset on children (Figure 1e). No differences were observed when data from the RV group on days 7 and 14 post-infection were compared (*p* > 0.9999, Figure 1h).

The differences regarding the expression of all the genes associated with the TREM-1 pathway were also investigated with the GSE41594 and GSE69529 datasets. Initially, the expression values of the genes were used to generate heatmaps. As seen in Figure 1i,j, different patterns of gene expressions were observed between healthy and infected individuals. For the child dataset, the pattern was more subtle, but there were several genes notably increased in children on days 1–3 post-infection (RV days 1–3), including *TREM1* and its adaptor protein, DAP12 (*TYROBP* gene), many pattern recognition receptors, transcription factors, pro-inflammatory cytokines, and caspases (highlighted in red, Figure 1i). On the other hand, in mice, the different patterns were very noticeable, with an overall peak in the TREM-1 pathway appearing on day 7, especially for caspases, chemokines, co-stimulatory proteins, integrins, and TLR genes, although *Trem1* itself peaked on day 3 (Figure 1j). The expression values of these genes were also used to generate PCA graphs. For the children’s dataset, we observed the overlap of the healthy and “RV 4–8” group (patients on days 4–8 after disease onset) but an overall distance between the “RV 1–3” group and both the healthy and “RV 4–8” groups, indicating that *TREM1* expression is time dependent and predominantly higher during the first three days of infection (Figure 1k). For the murine dataset, the clustering was very noticeable, indicating that the expression pattern of TREM-1 pathway genes was very different for each of the six groups (controls on days 3, 7, and 14, and RV-infected individuals on days 3, 7, and 14) (Figure 1l).

In summary, the transcriptomic data showed an increased expression of *TREM1*/*Trem1* and the genes associated with its activation during RV infection, regardless of the host’s nature (murine or human). Therefore, the receptor is likely activated and performs a biological response in this viral infection.

### 3.2. TREM-1 Inhibition Decreases the Production of Pro-Inflammatory Cytokines

**Figure 2:**
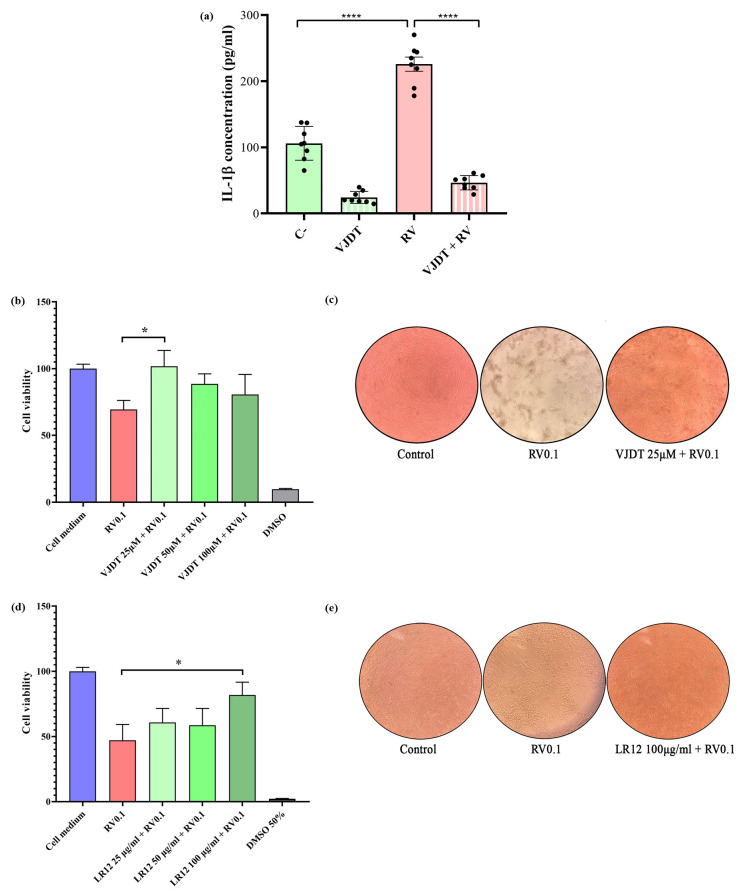
TREM-1 inhibition in vitro reduces the production of IL-1β and cytopathic effect during RVA infection. (**a**) IL-1β concentrations were assessed in the cell culture supernatant of THP-1 cells, with or without pre-treatment for 1 h with the TREM-1 inhibitor VJDT at 25 µM, followed by infection with RV at MOI 5, for 24 h. Concentrations (pg/mL) were assessed by ELISA. (**b**) Viability of MA104 cells (MTT assay) with or without treatment with VJDT at different concentrations (25 [pre-treatment plus post-treatment with 12.5 µM], 50 [pre-treatment plus post-treatment with 25 µM], and 100 µM [pre-treatment plus post-treatment with 50 µM]), followed by infection with RV at MOI 0.1, for 5 days (120 h). (**c**) Optical microscopy of MA104 cells after 5 days of incubation without RV infection (left), with RV infection at MOI 0.1 (middle), and with treatment with VJDT at 25 µM followed by RV infection (MOI 0.1, right). (**d**) Viability of MA104 cells (MTT assay) with or without pre-treatment with LR12 at different concentrations (25, 50, and 100 µg/mL), followed by infection with RV at MOI 0.1, for 5 days (120 h). DMSO at 50% was used as positive control of cell death. (**e**) Optical microscopy of MA104 cells after 5 days of incubation without RV infection (left), with RV infection at MOI 0.1 (middle), and with 1-h pre-treatment with LR12 at 100 µg/mL followed by RV infection (MOI 0.1, right). Statistical analyses conducted with one-way ANOVA followed by Turkey’s test (cytokine concentration) and Student’s *t*-test (cell viability). The column bar graphs depict mean values with the standard error of the mean (SEM), **** *p* < 0.0001, * *p* < 0.05.

To test whether TREM-1 could contribute to RV-related inflammatory responses, THP-1 monocytes were pre-treated for an hour with 25 µM of the TREM-1 inhibitor VJDT. This concentration was previously assessed by cell viability assays (Figure S1a). Then, THP-1 cells were infected with RV at a MOI of 5. After 24h of incubation, the supernatants were collected and the levels of IL-1β and TNF-α were measured by ELISA. RV infection induced the production of significant levels of IL-1β, with a 5-fold reduction observed with the use of the TREM-1 inhibitor (Turkey’s test *p* < 0.0001) (Figure 2a). THP-1 stimulated with LPS, a known TREM-1 activator, was used as a positive control (Figure S1b). Likewise, the receptor’s inhibition reduced the production of IL-1β (Turkey’s test *p* < 0.0001, Figure S1b). However, regarding TNF-α RV did not elicit the production of this cytokine to levels that were statistically different to the control, although the effect of VJDT in the drastic decrease in cytokine production was observed (Figure S1c). Therefore, our results suggest a contribution of TREM-1 to RV-related IL-1β-induced inflammation. 

### 3.3. TREM-1 Inhibition Decreases RV-Induced Cytopathic Effects

To test whether TREM-1 might impact the course of RV infection, the cytopathic effect was assessed through an MTT assay. For this analysis, MA104 cells were infected with RV at MOI 0.1, with a pre- and post-treatment with different concentrations of the TREM-1 inhibitor VJDT (Figure 2c,d). All the concentrations used were previously assessed by an MTT assay, which indicated their non-toxicity (Figure S2a). After 5 days of infection, the MTT assay was performed and indicated an increased cell viability when TREM-1 was inhibited with VJDT at 25 µM (pre- and post-treatment with 12.5 µM) (Figure 2c). Then, a different TREM-1 inhibitor, the LR12 peptide, was used for the same analysis, although this time we only conducted a pre-treatment (Figure 2d). Once again, after 5 days of incubation, the MTT assay indicated an increased cell viability when LR12 was used at 100 µg/mL (previously shown as non-toxic, Figure S2b) prior to the RV infection (Figure 2e). The positive effects of TREM-1 inhibition towards cytopathic effects (i.e., decreased cell lysis), independent of inhibition strategy, was also observed by optical microscopical analysis (Figure 2c,e), reinforcing the likely contribution of the receptor to RV infection and pathogenesis and to the host–virus relationship.

### 3.4. In Silico Simulations of the Interactions Between TREM-1 and RV Structural Proteins

As TREM-1 seemed to play a crucial role in RV infection, we used in silico tools to further investigate which viral structures could be recognized by the receptor. The PDB files corresponding to the IgV-like portion of human TREM-1, in addition to the capsomeres of RV TLP and DLP, were retrieved from the RCSB PDB database (1SMO, 4V7Q, and 3KZ4, respectively). The 1SMO structure was obtained by X-ray diffraction and had a resolution of 1.47 Å [89], while both 4V7Q (simian RRV strain) and 3KZ4 (bovine UK/G6 strain) were obtained by electron microscopy and had resolutions of 3.8 Å [11,12].

**Figure 3:**
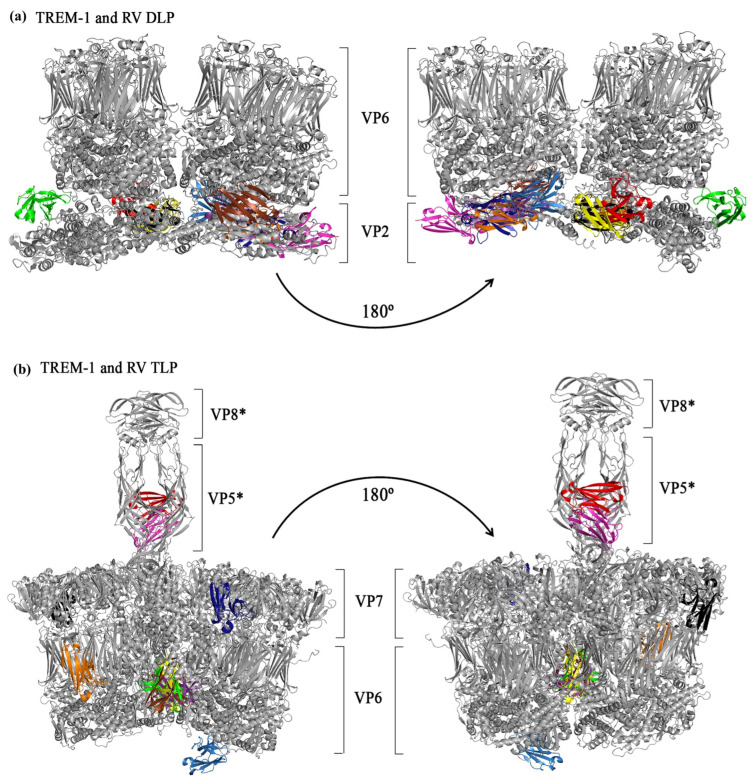
Molecular dockings between TREM-1 and capsomers of rotavirus (RV) double-layered particles and triple-layered particles. (**a**) Representations of the 10 best-ranked TREM-1/DLPs docked complexes. DLPs capsomere in gray (VP6 and VP2) with TREM-1 appearing in different colors (each color representing the position of TREM-1 in a different complex). (**b**) Representations of the 10 best-ranked TREM-1/TLPs docked complexes. TLPs capsomere in gray (only showing VP6, VP7, VP5*, and VP8*) with TREM-1 appearing in different colors (each color representing the position of TREM-1 in a different complex).

The molecular docking simulations were conducted in the ClusPro webserver and, comparing the 10 best ranked poses, TREM-1 did not interact with the surface portions of DLPs (formed by VP6 proteins), only the innermost part, which was not exposed in the viral particle for putative interactions with cell surface receptors (Figure 3a), thus suggesting that an interaction between TREM-1 and DLP proteins is unlikely to occur. On the other hand, with TLPs, the interactions occurred only with the stalk portion of the spike protein (corresponding to the VP5Ag domain of the VP5* subunit) (Figure 3b). Notably, the best ranked pose from the docking between TREM-1 and the TLP capsomere (Figure 3b, TREM-1 in red) showed an interaction with the receptor and the VP5Ag portions of the spike, suggesting that an interaction between TREM-1 and VP5*, specifically the VP5Ag domains, might occur.

To further explore the putative interaction between TREM-1 and VP5*, we conducted molecular docking simulations between TREM-1 and the VP5Ag domain from four different RV genotypes (P[4], P[8], and P[9]). The VP5* proteins were modeled with AlphaFold2 (via ColabFold) with sequences retrieved from GenBank (IDs: BBA27072.1 (P[4]), BBE28644.1 (P[8]), ATI14959.1 (P[9]), and ABH10616.1 (SA-11), aa 248–479 of VP4). The predicted structures (Figure S3a) were assessed with the Molprobity web server, which indicated that they had structural quality, represented by Molprobity score values lower than 2, and the majority of residues presented at energetically favorable regions (Table 1, Figure S3b–e). The known interaction [72] between VP5* (from the SA-11 RV strain) and ITGA4 (from the AlphaFold database, ID: AF-P13612-F1-v4) was used as a positive control (Figure S4a).

**Figure 4:**
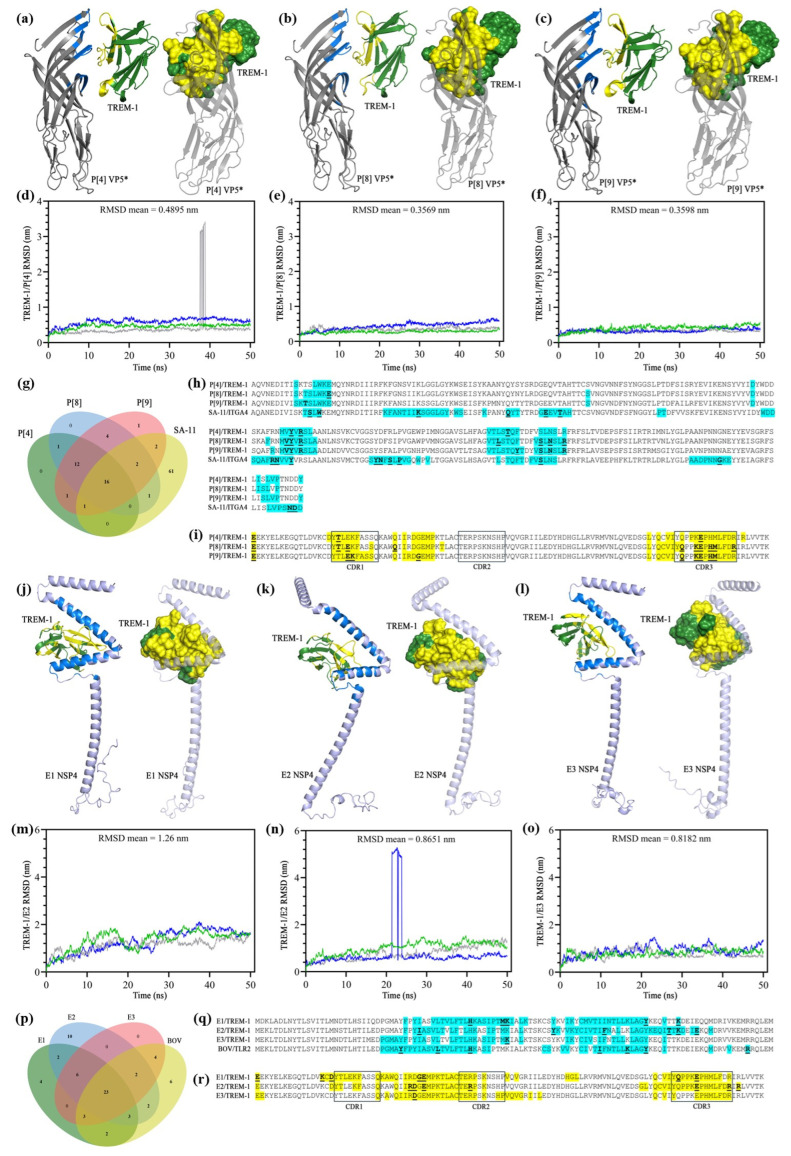
Molecular docking and molecular dynamics simulations between TREM-1 and rotavirus (RV) proteins. Selected docked poses of TREM-1 with the VP5Ag domain of P[4] (**a**), P[8] (**b**), and P[9] (**c**) genotypes of the VP5* protein. TREM-1 is depicted in green, with interacting residues in yellow, while VP5* is depicted in gray, with interacting residues in blue. Graphs displaying the root mean square deviation (RMSD) values throughout the 50-nanosecond (ns) molecular dynamics simulations between TREM-1 and P[4] (**d**), P[8] (**e**), and P[9] (**f**) genotypes of the VP5* protein. Each blue, green, and gray line represents a replicate. (**g**) Venn diagram for the VP5* residues from the P[4] (green), P[8] (blue), P[9] (red), and SA-11 (yellow) genotypes which are recognized by TREM-1. (**h**) Amino acid sequences of the VP5* structures used in the molecular docking and dynamics simulations, with TREM-1-interacting residues highlighted in cyan, and those mediating hydrogen bonds in bold and underlined. (**i**) Amino acid sequences of the TREM-1 structure used in the simulations, with VP5*-interacting residues highlighted in yellow, and those mediating hydrogen bonds in bold and underlined. Selected docked poses of TREM-1 with the NSP4 proteins from the E1 (**j**), E2 (**k**), and E3 (**l**) genotypes. TREM-1 is depicted in green, with interacting residues in yellow, while NSP4 is depicted in lilac, with interacting residues in blue. Graphs displaying the root mean square deviation (RMSD) values throughout the 50-nanosecond (ns) molecular dynamics simulations between TREM-1 and the E1 (**m**), E2 (**n**), and E3 (**o**) genotypes of the NSP4 protein. Each blue, green, and gray line represents a replicate. (**p**) Venn diagram for the NSP4 residues from the E1, E2, E3, and bovine genotypes recognized by TREM-1. (**q**) Amino acid sequences of the NSP4 structures used in the molecular docking and dynamics simulations, with TREM-1-interacting residues highlighted in cyan, and those mediating hydrogen bonds in bold and underlined. (**r**) Amino acid sequences of the TREM-1 structure used in the simulations, with NSP4 interacting residues highlighted in yellow, and those mediating hydrogen bonds in bold and underlined.

After the selection of the poses (Figure 4a–c), based on the “lowest energy” score of Cluspro, MD simulations were conducted for 50 ns. The RMSD values of all the three TREM-1/VP5Ag complexes remained stable throughout time, without the presence of large peaks, presenting the following RMSD means: 0.4895nm for TREM-1/P[4], 0.3569 nm for TREM-1/P[8], and 0.3598nm for TREM-1/P[9] (Figure 4d–f). These results indicate the stability of the predicted interactions between TREM-1 and the different genotypes of VP5*. In addition, analysis with the PIZSA web server also indicated the binding of VP5Ag to TREM-1, as Z-score values representing stable interactions (>1.000) were found for all complexes (1.802, 1.442, and 1.757 for P[4], P[8], and P[9], respectively) (Table 1). The VP5Ag/ITGA4 pose also showed stability in the MD simulations (RMSD mean: 0.5916 nm) (Figure S3a) and a high Z-score (1.547), indicating binding between the two proteins, thus validating our in silico protocol.

The energetic parameters of the TREM-1/VP5Ag complexes were further assessed (Table 1). Analysis conducted in the CCharPPI webserver resulted in values within the range observed for other protein–protein complexes, thus reinforcing the likelihood of such complexes to occur in vivo. We also predicted the binding free energy and dissociation constants of the complexes, observing that TREM-1/P[9] had the highest values for these parameters amongst the TREM-1/VP5 complexes, suggesting a genotype-based difference regarding the affinity of interactions with TREM-1.

The interaction interfaces of the VP5Ag/TREM-1 complexes were defined with the combination of results obtained with analyses on PPCheck, PDBSum, and DIMPLOT, and are depicted in 4a to 4c (interacting residues are highlighted in blue [VP5Ag] and yellow [TREM-1]). We found a remarkable similarity regarding the VP5* residues and the different genotypes which were recognized by TREM-1, which can be observed in Figure 4h (highlighted in cyan) and in the Venn diagram (Figure 4g). In addition, most of the recognized VP5* regions were conserved between the three genotypes; therefore, our results are in accordance with the pattern expected from interactions between a viral protein and its coreceptor. Furthermore, TREM-1 interacted with the viral residues mostly at its C and C’ β-strands, in addition to the CDR1 (complementarity determining region) and CDR3 regions, which are known as the main antigen recognition sites within the receptor [90]. Altogether, our data suggests that TREM-1 might recognize RV TLPs at the VP5Ag domain of the viral spike, probably acting as a coreceptor for viral entry, which may explain the findings observed in vitro, with the inhibition of the receptor resulting in a less cytopathic effect. 

### 3.5. In Silico Simulations of Interactions Between TREM-1 and the RV Enterotoxin

Considering the importance of RV NSP4 as an enterotoxin and its description as a pathogen-associated molecular pattern (PAMP) binding to TLR2 [71], we further investigated whether an interaction between this protein and TREM-1 would also occur. Therefore, we predicted the structure of NSP4 proteins from three different genotypes (E1, E2, and E3) using AlphaFold2. The interaction between NSP4 and TLR2 was used as a control in accordance with the experiments conducted by Ge et al. (2013) [71]. The NSP4 structure was modeled by AlphaFold2 as well (with the sequence obtained from a UK bovine strain, Genbank ID: K03384.1) (Figure S3f), while TLR2 was retrieved from the RCS PDB (ID: 2Z7X) [91]. Molprobity analysis indicated the structural quality of the viral proteins, with values close to 2 being observed (Table 2) and with the presence of the majority of the residues within energetically favorable regions in the Ramachandran plot (Figure S3g–j).

Following the molecular docking simulations on Cluspro, the poses were selected (Figure 4j–l) and submitted to molecular dynamics to assess their stability. For these simulations, we only maintained the NSP4 amino acids close to the interaction interface (aa 26–134) in an attempt to minimize the contribution of flexible regions outside the recognized domain to the final RMSD value. In general, an absence of peaks was observed (Figure 4m–o), indicating the stability of the complex, although the overall RMSD values were considered high: the RMSD means for TREM-1/E1, TREM-1/E2, and TREM-1/E3 were 1.26 nm, 0.8651 nm, and 0.8182 nm, respectively. However, when the RMSF values were analyzed (Figure S5d–f), we found that the residues predicted to be part of the interaction interfaces had lower values (close to 0.5 nm), thus it is likely that the highly flexible structure of NSP4 might have contributed to the higher values of the RMSDs. Regarding the control interaction between NSP4 and TLR2 (Figure S5a–c), a similar pattern to the NSP4/TREM-1 interactions was observed in the molecular dynamics simulations, with an absence of peaks in the 50 ns simulations but an RMSD of 0.9347 nm. The protein complexes were then analyzed in the PIZSA webserver, which indicated that all the NSP4/TREM-1 complexes had stable interactions, as well as the NSP4/TLR2 complex, which validates our protocol (Table 2). A genotype-dependent difference was, once again, observed, with the TREM-1/E2 complex showing the highest Z-score, 1.950, while TREM-1/E1 had the lowest value of 1.162 (Table 2).

Further energetic analyses were conducted with the TREM-1/NSP4 complexes (Table 2). Reinforcing the likelihood of these complexes existing, the results were within the range shown by other protein–protein complexes. Comparing binding free energy and dissociation constants, the values varied from −76.66 kcal/mol (TREM-1/E1) to −59.38 kcal/mol (TREM-1/E3) and from 2.4 × 10^−9^ (TREM-1/E2) to 1.4 × 10^−8^ (TREM-1/E1), respectively. Taken together with the PIZSA score, these results suggest that TREM-1 might have a higher affinity for the E2 NSP4 protein, which could lead to different infection outcomes or virulence, although in vitro analysis conducted with different viral strains is required to fully investigate this.

The interaction interfaces of the TREM-1/NSP4 complexes were also defined with the combination of results from PPCheck, PDBSum generate, and DIMPLOT. The 3D structures of the complexes are shown in Figure 4j–l, with the interacting residues highlighted in blue (NSP4) and yellow (TREM-1). A remarkable similarity regarding the recognized regions in NSP4 was observed, which can also be seen in the Venn graph at Figure 4p–r, where the protein sequences are displayed. The NSP4 residues recognized by TREM-1 are concentrated in the 28–99 region, which encompasses the hydrophobic and viroporin domains of this protein, as well as the beginning of the coiled domain [26]. The same regions were also recognized by TLR2 (Figure 4q), a pattern that reinforces the likelihood that TREM-1 acts as a pattern recognition receptor (PRR) in RV infection. In addition, the main regions of TREM-1 mediating this interaction (C and C’ strands, CDR1, CDR2, and CDR3) have already been described as important for antigen recognition [90].

## 4. Discussion

In the present study, we used different approaches to investigate the involvement of TREM-1 in RV infection and pathogenesis, which has not been addressed before. Initially, we searched for public transcriptomic data to observe whether the TREM-1 pathway was activated in RV infection. TREM-1 and its associated genes were upregulated in infected mice and humans, mainly during the early stages of infection, although the receptor was not associated with the disease severity. Next, we investigated cytokine production by a human monocyte lineage exposed to RV with or without TREM-1 inhibition. The abrogation of the receptor’s activity resulted in reduced production of pro-inflammatory cytokines. Also, this inhibition was associated with reduced cytopathic effects in infected MA104 cells. To predict which viral proteins were recognized by TREM-1, we used different in silico tools, which suggested interactions between the receptor with the VP5* protein. This result could at least partly explain the reduction in cytopathic effects observed in vitro when the receptor’s activity was abrogated. In addition, our in silico analysis also suggested an interaction between TREM-1 and NSP4, an enterotoxin with immunostimulant properties, which might be involved in the cytokine production process.

TREM-1 was initially described as an amplifier of inflammatory responses in the 2000s [50]. Its activation has been described in a variety of diseases, where the receptor has been associated with cytokine and chemokine production, including IL-1β, TNF-α, IL-6, IL-8, CCL2, CCL7, and CCL3 [50,56,59,60,61,62], and as an inducer of neutrophil degranulation, respiratory burst, and NETosis [61,92,93]. Within viral infections, TREM-1 has been associated with the production of pro-inflammatory cytokines [44,46,94,95,96], the inhibition of apoptosis in macrophages and microglia infected with HIV [43,48]; severe outcomes in hand, foot, and mouth disease (caused by enterovirus A71) [42]; progressive liver damage and cirrhosis onset in hepatitis B infection [96,97]; and hyperinflammation and severity of COVID-19 [98,99,100,101], among others. Recently, our group investigated the role of the receptor in norovirus (NV) infection, another etiologic agent of DD [102]. Using transcriptomic data analysis, we observed the increased expression of the receptor in murine macrophages exposed to the murine strain of norovirus (MNoV), as well as the coexpression of TREM-1, cytokines, and PRRs (including IL-1β, TNF-α, TLR2, TLR4, and TRL8), in addition to pyroptosis-associated genes, suggesting the involvement of the receptor in the viral pathogenesis. Additionally, molecular dockings and dynamics simulations indicated stable interactions between TREM-1 and the viral protein VP1, suggesting a role for the receptor in the host–pathogen relationship, possibly as a coreceptor [102].

Considering the role of TREM-1 in gastroenteric inflammatory diseases, such as in inflammatory bowel disease [103], celiac disease [104], and NV infection, we hypothesized that the receptor might play a role in RV infection. With the analysis of transcriptomic data, we found the receptor significantly increased in two different studies, using data from experimentally infected mice and children with DD. TREM-1 seems to be associated with the early stages of RV infection, with its expression decreasing throughout time, which is in accordance with previous studies conducted on viral infections (dengue and Crimean–Congo hemorrhagic fever) [105,106]. This finding can be at least partially explained by TREM-1 expression and activation kinetics: with the activation of a PRR, such as TLRs, the expression of TREM-1 is upregulated and the receptor recognizes its ligands, resulting in the activation of its signaling pathway [107]. However, a hallmark of TREM-1 activation is the expression of matrix metalloproteinases (MMP), which promotes the cleavage of the membrane-associated form of the receptor, resulting in the release of sTREM-1 (soluble TREM-1), which acts as a decoy molecule, blocking the signaling process associated with membrane-associated TREM-1 [108,109]. The peak of TREM-1 expression in monocytes, following LPS administration, has been reported to occur between the first and second hours after exposure, with an increased presence on the membrane at 6 h, followed by a progressive decrease, concomitant to sTREM-1 accumulation, up to 24 h (maximum time assessed in the study) [110]. Thus, the progressive cleavage of the receptor on the cell membrane following its activation could explain the involvement of TREM-1 at the early stages of infections, as we observed in the present study. Furthermore, our data indicated no association between TREM-1 expression and disease severity, which has been reported in other infections [95,100,111,112,113]. However, this association cannot be ruled out, as methodological aspects might have influenced this result, such as number of samples, cell/tissue type, and the time of infection.

Besides the TREM-1 gene, we also investigated the expression of its entire signaling pathway (as used in QIAGEN Ingenuity Pathway Analysis software). Our results showed a clear difference regarding the expression pattern of the healthy/control groups compared to the infected mice and children. Notably, we found the concomitant upregulation of different TLRs and NLRs, chemokines, and cytokines with *TREM1*/*Trem1*, which is expected for an amplifier of pro-inflammatory responses. Amongst the upregulated cytokines are TNF-α and IL-1β. These two pro-inflammatory molecules are commonly associated with TREM-1 activation [50,56,59,62], and have been detected in association with RV infection in clinical, in vivo, and in vitro studies, with TNF-α being correlated to the occurrence of fever and higher frequency of diarrhea, but also to anti-viral activity against RV [114,115,116,117,118,119,120,121,122]. In our in vitro experiments with THP-1 cells, which are cells from a human monocyte cell lineage, we observed the production of IL-1β in response to RV infection, with the inhibition of TREM-1 resulting in reductions in this cytokine to similar levels as those observed in uninfected controls. Although the precise roles of IL-1β and TNF-α in RV infection have not been fully investigated, they are (together with IL-6) the traditional cytokines associated with acute inflammation, promoting the stimulation of T and B cells, the production of ROS and NO, neutrophilia, and the secretion of chemokines and acute-phase proteins in the liver [123,124]. They can also lead to tissue damage, being commonly associated with the pathology of several chronic inflammatory diseases, such as IBD [125,126]. Furthermore, IL-1β and TNF-α are known to cause fever, and TNF-α is associated with the development of secretory diarrhea, which are two hallmarks of RV pathology [127,128,129,130]. Therefore, as TREM-1 seems crucial for the production of a pro-inflammatory cytokine, it is possible that the receptor might be implicated in the physiopathology of RV infection.

We also investigated how the inhibition of TREM-1 would impact the development of the RV infection in an epithelial cell lineage, MA104, which is highly susceptible to the RRV infection. Surprisingly, the inhibition of TREM-1 resulted in decreased cytopathic effects, which is directly associated with viral replication. This result indicates that TREM-1 might be important to the viral infection, possibly acting as a coreceptor for RV entry. RV adhesion to target cells is mainly mediated by the VP4 protein, which is proteolytically cleaved into two sub-domains, VP5* and VP8*. VP8* mediates the initial interaction with the host cells through binding to different glycans, such as sialic acid and human histo-blood group antigen (HBGA) [131,132]. VP5* and VP7 are thought to act after VP8*, interacting with different protein targets, such as integrins and the heat shock cognate 71 kDa protein (Hsc70) to promote viral entry [131,133]. Whether these interactions occur sequentially is not well known, but it has been shown that not all viral strains require integrins for binding, but they all require Hsc70, dynamin, and cholesterol for efficient infection [133,134].

In an attempt to predict whether TREM-1 could act as a coreceptor, which could justify the findings regarding the cytopathic effects, we performed molecular docking and dynamics simulations. Initially, we simulated interactions among TREM-1 and capsomers from the TLP and DLP RV structures. This approach indicated an interaction between TREM-1 and the VP5Ag domain of VP5*, which led us to simulate the interaction of the receptor to different viral genotypes of VP5*. The complexes were predicted as stable, sharing not only considerable similarities regarding the interaction interfaces between themselves but also to the known VP5/ITGA4 interaction, reinforcing the likelihood of the TREM-1/VP5* interaction ocurring in nature. Heightening the possibility of TREM-1 being a coreceptor in RV infection is the fact that, upon its activation, TREM-1 has been detected in ganglioside M1-lipid rafts [92], which are important structures for the entry and exit of RV from host cells [135,136,137]. Lipid rafts are ordered structures within cellular membranes that are detergent insoluble and rich in phospholipids and cholesterol where different proteins can be clustered, facilitating signaling processes [138]. Initial studies showed that the depletion of cholesterol in cell membranes significantly reduced the infectivity of RV, with further analyses showing the co-localization of ganglioside GM1, integrins, and Hsc70 in detergent-resistant domains from MA104 cells [16,139,140]. Importantly, when the known cellular targets of RV were blocked by different approaches, the infectivity of the virus was not completely abolished, indicating that undescribed RV coreceptors are likely to exist. Here, we hypothesize that TREM-1 might play a role as a coreceptor, possibly being recruited into lipid rafts, where it can work together with Hsc70 and integrins in the promotion of RV entry.

Finally, we investigated whether TREM-1 could interact with NSP4. This protein has multiple roles during RV infection, being important for viroplasm formation and viral assembly, but also acting as an enterotoxin, being directly implicated in diarrhea induction [7,26]. Previous studies showed that peptides derived from the 114–135 domain of NSP4 were able to induce diarrhea in young mice [141]. Later, NSP4 has been described as an immunogenic protein [26,71]. In this context, TLR2 has been associated with NSP4 recognition, resulting in the release of IL-6 and TNF-α [71]. Considering the importance of TREM-1 to the production of inflammatory cytokines during RV infection, including TNF-α, which we observed here, beyond the known collaboration between TREM-1 and TLR2 [56,61,142], interactions between TREM-1 and different genotypes of NSP4 were simulated. Our results indicated an interaction between these proteins and TREM-1, occurring mainly in the viroporin domain of NSP4, which is highly conserved in the RVA genotypes [26]. Although studies have shown the 114–135 region as the secreted enterotoxic domain [141,143], mutations in residue 37 (present in the interactions observed in our results) were associated with virus virulence and development of diarrhea [144]. Due to limitations regarding the obtention of viable viruses with NSP4 mutations and in the production of recombinant NSP4 proteins, there are still many gaps to be filled regarding the regions associated with the biological function of this protein. Nonetheless, our in silico and in vitro results indicate TREM-1 as a possible receptor for NSP4.

Our study has some limitations, including the use of a non-purified viral stock, which might have contained proteins that can act as TREM-1 ligands as well, and the use of in silico simulations, which are unable to fully replicate the dynamic processes of protein–protein and virion–coreceptor interactions. Future studies conducted with purified RV particles and proteins using different in vitro and in vivo strategies could confirm our predicted interactions between RV proteins and TREM-1. Nevertheless, the combination of the transcriptomic, in vitro, and in silico results presented here strongly suggest that TREM-1 might be an important player in RV infection and pathogenesis, possibly acting in the physiopathology of this infection through the recognition of viral proteins, like the NSP4 enterotoxin, and as a coreceptor for the cell entry process, thus being a promising biomarker candidate for infection diagnosis and prognosis and a target for therapeutic interventions.

## Figures and Tables

**Table 1:** Interaction of different genotypes of VP5 with TREM-1 and HSPA8.

**Protein Modeling**				
Genotypes	P[4]	P[8]	P[9]	SA-11
Genbank ID	BBA27072.1	BBE28644.1	ATI14959.1	ABH10616.1
Amino acids	248–479	248–479	248–479	247–479
Molprobity score	0.87	1.0	1.24	1.15
Clashscore	1.37	2.2	4.14	2.47
Poor rotamers	0.49%	0.49%	0.98%	0.99%
Favored rotamers	99.02%	99.02%	98.53%	97.03%
Ramachandran outliers	0.43%	0.43%	0.43%	0.43%
Ramachandran favored	98.27%	98.27%	97.84%	97.39%
Bad bonds	0%	0%	0%	0%
Bad angles	0.19%	0.23%	0.16%	0.27%
** Molecular Docking **				
Complex	P[4]/TREM-1	P[8]/TREM-1	P[9]/TREM-1	SA-11/ITGA4
ΔG (kcal/mol)	−46.2	−67.8	−73.0	−112.0
Kd (M) (36.5 °C)	2.3 × 10^−7^	2.3 × 10^−7^	2.8 × 10^−8^	4 × 10^−15^
PYDOCK_TOT (−60 a −5)	−47.957	−42.631	−36.571	−66.499
ELE (−60 a 0)	−13.13	−16.422	−16.433	−15.808
HBOND (−15 a −1)	−4.37	−8.55	−5.03	0.0
VDW (−200 a −50)	−78.941	−89.175	−104.721	−185.153
FA_ATR (−100 a −20)	−43.377	−56.54	−62.045	−125.133
DESOLV (−30 a 20)	−26.932	−17.291	−9.666	−32.176
Z-score (binder: >1.000)	1.802	1.442	1.757	1.547

ΔG—Gibbs free energy; Kd—dissociation constant; PYDOCK_TOT—total energy; HBOND—hydrogen bond potential; VDW—van der Waals energy; ELE—total electrostatic energy; FA_ATR—attractive van der Waals forces; DESOLV—desolvation energy.

**Table 2:** Interaction of different genotypes of NSP4 with TREM-1.

**Protein Modeling**				
Genotypes	E1	E2	E3	BOV
Genbank ID	AYJ18795.1	AWH62678.1	ALJ03222.1	K03384.1
Molprobity score	2.17	1.86	2.00	2.17
Clashscore	3.46	2.07	3.77	2.4
Poor rotamers	4.24%	3.09%	3.03%	5.49%
Favored rotamers	90.91%	90.12%	92.12%	90.85%
Ramachandran outliers	2.89%	2.31%	2.89%	3.47%
Ramachandran favored	89.60%	91.33%	92.49%	88.44%
Bad bonds	0%	0%	0%	0%
Bad angles	0.36%	0.57%	0.41%	0.26%
** Molecular Docking **				
Complex	E1/TREM-1	E2/TREM-1	E3/TREM-1	BOV/TLR2
ΔG (kcal/mol)	−76.66	−74.79	−59.38	−42.15
Kd (M) (36.5 °C)	1.4 × 10^−8^	2.4 × 10^−9^	9.6 × 10^−8^	3.8 × 10^−11^
PYDOCK_TOT (−60 a −5)	−45.782	−48.499	−60.849	−54.45
ELE (−60 a 0)	−17.75	−16.016	−7.766	−6.262
HBOND (−15 a −1)	−3.83	−5.38	−1.81	−6.0
VDW (−200 a −50)	−114.961	−119.72	−97.915	−128.306
FA_ATR (−100 a −20)	−70.462	−75.283	−51.673	−71.156
DESOLV (−30 a 20)	−16.536	−20.511	−43.291	−35.358
Z-score (binder > 1.000)	1.162	1.950	1.750	1.900

ΔG—Gibbs free energy; Kd—dissociation constant; PYDOCK_TOT—total energy; HBOND—hydrogen bond potential; VDW—van der Waals energy; ELE—total electrostatic energy; FA_ATR—attractive van der Waals forces; DESOLV—desolvation energy.

## Data Availability

The data presented in this study are available in QIAGEN gene dataset—https://geneglobe.qiagen.com/us/knowledge/pathways/trem1-signaling; Gene Expression Omnibus (GEO, at https://www.ncbi.nlm.nih.gov/geo/, reference numbers: GSE41594, GSE50628, GSE69529 and GSE41594 (RCS-PDB at https://www.rcsb.org, reference numbers: 1SMO (TREM-1), 4V7Q (RV TLP), 3KZ4 (RV DLP), and 2Z7X (TLR2); GenBank at https://www.ncbi.nlm.nih.gov/genbank/, reference numbers: K03384.1 (RV NSP4); AYJ18795.1 (RV NSP4 E1); AWH62678.1 (RV NSP4 E2); ALJ03222.1 (RV NSP4 E3); BBA27072.1 (P[4]), BBE28644.1 (P[8]), ATI14959.1 (P[9]), and ABH10616.1 (SA-11), aa 248–479 of VP4); AlphaFold database at https://alphafold.ebi.ac.uk/, reference number: AF-P13612-F1-v4 (all accessed on 15 September 2025).

## Supplementary Materials

The following supporting information can be downloaded at: https://www.mdpi.com/article/10.3390/pathogens14101029/s1, Figure S1: TREM-1 inhibition impact in cytokine production. Figure S2: evaluation of the toxicity of TREM-1 inhibitors in MA104 cells. Figure S3: structure prediction of VP5* and NSP4 proteins. Figure S4: interaction between SA-11 VP5* and integrin alpha 4 (ITGA4). Figure S5: interaction between bovine NSP4 and toll-like receptor 2 (TLR2).

## Abbreviations

The following abbreviations are used in this manuscript:

DD	Diarrheal disease
AEBSF	4-(2-aminoethyl)benzenesulfonyl fluoride hydrochloride
AG	Acute
CT	Control
DEGs	Differentially expressed genes
DESOLV	Desolvation energy
DLP	Double-layered particle
DMSO	Dimethyl sulfoxide
ELE	Total electrostatic energy
ELISA	Enzyme-linked immunosorbent assay
FA_ATR	Attractive van der Waals forces
HBOND	Hydrogen bond potential
ICTV	International Committee on Taxonomy of Viruses
ISGs	Interferon-stimulated genes
Kd	Dissociation constant
LPS	Lipopolysaccharide
MD	Molecular dynamics
MM/GBSA	Molecular Mechanics–Generalized Born Surface Area
MOI	Multiplicity of infection
MTT	3-(4,5-dimethylthiazol-2-yl)-2,5-diphenyl-2H-tetrazolium bromide
PCA	Principal component analysis
PFU	Plaque-forming units
PYDOCK_TOT	Total energy
RE	Recovery
RMSD	Root mean square deviation
RV	Rotavirus
RVA	Rotavirus A
SE	Seizure
sTREM-1	Soluble triggering receptor expressed on myeloid cells 1
TCID50	50% tissue culture infectious dose
TLP	Triple-layered particle
TREM-1	Triggering receptor expressed on myeloid cells 1
VDW	Van der Waals energy
ΔG	Gibbs free energy

## References

[1] Kyu H.H., Vongpradith A., Dominguez R.-M.V., Ma J., Albertson S.B., Novotney A., Khalil I.A., Troeger C.E., Doxey M.C., Ledesma J.R. (2025). Global, regional, and national age-sex-specific burden of diarrhoeal diseases, their risk factors, and aetiologies, 1990–2021, for 204 countries and territories: A systematic analysis for the Global Burden of Disease Study 2021. Lancet Infect. Dis..

[2] Vos T., Lim S.S., Abbafati C., Abbas K.M., Abbasi M., Abbasifard M., Abbasi-Kangevari M., Abbastabar H., Abd-Allah F., Abdelalim A. (2020). Global burden of 369 diseases and injuries in 204 countries and territories, 1990–2019: A systematic analysis for the Global Burden of Disease Study 2019. Lancet.

[3] Bányai K., Estes M.K., Martella V., Parashar U.D. (2018). Viral gastroenteritis. Lancet.

[4] Burnett E., Jonesteller C.L., Tate J.E., Yen C., Parashar U.D. (2017). Global Impact of Rotavirus Vaccination on Childhood Hospitalizations and Mortality From Diarrhea. J. Infect. Dis..

[5] Tate J.E., Burton A.H., Boschi-Pinto C., Parashar U.D. (2016). Global, Regional, and National Estimates of Rotavirus Mortality in Children <5 Years of Age, 2000–2013. Clin. Infect. Dis..

[6] Matthijnssens J., Ciarlet M., McDonald S.M., Attoui H., Bányai K., Brister J.R., Buesa J., Esona M.D., Estes M.K., Gentsch J.R. (2011). Uniformity of rotavirus strain nomenclature proposed by the Rotavirus Classification Working Group (RCWG). Arch. Virol..

[7] Crawford S.E., Ding S., Greenberg H.B., Estes M.K., Knipe D.M., Howley P.M., Cohen J.I., Damania B., Enquist L., Freed E.O., Wheelan S.P.J. (2022). Rotaviruses. Fields Virology: RNA Viruses.

[8] International Committee on Taxonomy of Viruses (2025). Taxonomy Browser. https://ictv.global/taxonomy.

[9] Desselberger U. (2014). Rotaviruses. Virus Res..

[10] Mathieu M., Petitpas I., Navaza J., Lepault J., Kohli E., Pothier P., Prasad B.V., Cohen J., Rey F.A. (2001). Atomic structure of the major capsid protein of rotavirus: Implications for the architecture of the virion. EMBO J..

[11] McClain B., Settembre E., Temple B.R., Bellamy A.R., Harrison S.C. (2010). X-ray crystal structure of the rotavirus inner capsid particle at 3.8 A resolution. J. Mol. Biol..

[12] Settembre E.C., Chen J.Z., Dormitzer P.R., Grigorieff N., Harrison S.C. (2011). Atomic model of an infectious rotavirus particle. EMBO J..

[13] Shah P.N.M., Gilchrist J.B., Forsberg B.O., Burt A., Howe A., Mosalaganti S., Wan W., Radecke J., Chaban Y., Sutton G. (2023). Characterization of the rotavirus assembly pathway in situ using cryoelectron tomography. Cell Host Microbe.

[14] Ciarlet M., Crawford S.E., Cheng E., Blutt S.E., Rice D.A., Bergelson J.M., Estes M.K. (2002). VLA-2 (alpha2beta1) integrin promotes rotavirus entry into cells but is not necessary for rotavirus attachment. J. Virol..

[15] Isa P., Arias C.F., López S. (2006). Role of sialic acids in rotavirus infection. Glycoconj. J..

[16] Isa P., Realpe M., Romero P., López S., Arias C.F. (2004). Rotavirus RRV associates with lipid membrane microdomains during cell entry. Virology.

[17] Liu Y., Ramelot T.A., Huang P., Liu Y., Li Z., Feizi T., Zhong W., Wu F.T., Tan M., Kennedy M.A. (2016). Glycan Specificity of P [19] Rotavirus and Comparison with Those of Related P Genotypes. J. Virol..

[18] López S., Arias C.F. (2004). Multistep entry of rotavirus into cells: A Versaillesque dance. Trends Microbiol..

[19] Pang L.L., Wang M.X., Sun X.M., Yuan Y., Qing Y., Xin Y., Zhang J.Y., Li D.D., Duan Z.J. (2018). Glycan binding patterns of human rotavirus P [10] VP8* protein. Virol. J..

[20] Ramani S., Hu L., Venkataram Prasad B.V., Estes M.K. (2016). Diversity in Rotavirus-Host Glycan Interactions: A “Sweet” Spectrum. Cell. Mol. Gastroenterol. Hepatol..

[21] Venkataram Prasad B.V., Shanker S., Muhaxhiri Z., Choi J.M., Atmar R.L., Estes M.K., Svensson L., Desselberger U., Greenberg H.B., Estes M.K. (2016). Chapter 3.1—Structural Biology of Noroviruses. Viral Gastroenteritis.

[22] Yoder J.D., Trask S.D., Vo T.P., Binka M., Feng N., Harrison S.C., Greenberg H.B., Dormitzer P.R. (2009). VP5* rearranges when rotavirus uncoats. J. Virol..

[23] Yu X., Guillon A., Szyczew A.J., Kiefel M.J., Coulson B.S., von Itzstein M., Blanchard H. (2008). Crystallization and preliminary X-ray diffraction analysis of the carbohydrate-recognizing domain (VP8*) of bovine rotavirus strain NCDV. Struct. Biol. Cryst. Commun..

[24] WHO (2020). Rotavirus vaccines. The Immunological Basis for Immunization Series.

[25] Gómez-Rial J., Sánchez-Batán S., Rivero-Calle I., Pardo-Seco J., Martinón-Martínez J.M., Salas A., Martinón-Torres F. (2019). Rotavirus infection beyond the gut. Infect. Drug Resist..

[26] Sastri N.P., Crawford S.E., Estes M.K., Svensson L., Desselberger U., Greenberg H.B., Estes M.K. (2016). Chapter 2.4—Pleiotropic Properties of Rotavirus Nonstructural Protein 4 (NSP4) and Their Effects on Viral Replication and Pathogenesis. Viral Gastroenteritis.

[27] Mohammed F.S., Uysal İ., Sevindik M. (2023). A review on antiviral plants effective against different virus types. Prospect. Pharm. Sci..

[28] Jiang L., Tang A., Song L., Tong Y., Fan H. (2023). Advances in the development of antivirals for rotavirus infection. Front. Immunol..

[29] La Frazia S., Ciucci A., Arnoldi F., Coira M., Gianferretti P., Angelini M., Belardo G., Burrone O.R., Rossignol J.F., Santoro M.G. (2013). Thiazolides, a new class of antiviral agents effective against rotavirus infection, target viral morphogenesis, inhibiting viroplasm formation. J. Virol..

[30] Wang J., Hu X., Wu J., Lin X., Chen R., Lu C., Song X., Leng Q., Li Y., Kuang X. (2024). ML241 Antagonizes ERK 1/2 Activation and Inhibits Rotavirus Proliferation. Viruses.

[31] Bassetto M., Van Dycke J., Neyts J., Brancale A., Rocha-Pereira J. (2019). Targeting the Viral Polymerase of Diarrhea-Causing Viruses as a Strategy to Develop a Single Broad-Spectrum Antiviral Therapy. Viruses.

[32] Holloway G., Coulson B.S. (2013). Innate cellular responses to rotavirus infection. J. Gen. Virol..

[33] Angel J., Franco M.A., Greenberg H.B. (2012). Rotavirus immune responses and correlates of protection. Curr. Opin. Virol..

[34] Broquet A.H., Hirata Y., McAllister C.S., Kagnoff M.F. (2011). RIG-I/MDA5/MAVS are required to signal a protective IFN response in rotavirus-infected intestinal epithelium. J. Immunol..

[35] Schoggins J.W., MacDuff D.A., Imanaka N., Gainey M.D., Shrestha B., Eitson J.L., Mar K.B., Richardson R.B., Ratushny A.V., Litvak V. (2014). Pan-viral specificity of IFN-induced genes reveals new roles for cGAS in innate immunity. Nature.

[36] Sen A., Pruijssers A.J., Dermody T.S., García-Sastre A., Greenberg H.B. (2011). The early interferon response to rotavirus is regulated by PKR and depends on MAVS/IPS-1, RIG-I, MDA-5, and IRF3. J. Virol..

[37] Pott J., Stockinger S., Torow N., Smoczek A., Lindner C., McInerney G., Bäckhed F., Baumann U., Pabst O., Bleich A. (2012). Age-dependent TLR3 expression of the intestinal epithelium contributes to rotavirus susceptibility. PLoS Pathog..

[38] Zhu S., Ding S., Wang P., Wei Z., Pan W., Palm N.W., Yang Y., Yu H., Li H.B., Wang G. (2017). Nlrp9b inflammasome restricts rotavirus infection in intestinal epithelial cells. Nature.

[39] Dantas P., Matos A.O., da Silva Filho E., Silva-Sales M., Sales-Campos H. (2020). Triggering receptor expressed on myeloid cells-1 (TREM-1) as a therapeutic target in infectious and noninfectious disease: A critical review. Int. Rev. Immunol..

[40] de Oliveira Matos A., Dos Santos Dantas P.H., Figueira Marques Silva-Sales M., Sales-Campos H. (2020). The role of the triggering receptor expressed on myeloid cells-1 (TREM-1) in non-bacterial infections. Crit. Rev. Microbiol..

[41] Matos A.O., Dantas P., Silva-Sales M., Sales-Campos H. (2021). TREM-1 isoforms in bacterial infections: To immune modulation and beyond. Crit. Rev. Microbiol..

[42] Amrun S.N., Tan J.J.L., Rickett N.Y., Cox J.A., Lee B., Griffiths M.J., Solomon T., Perera D., Ooi M.H., Hiscox J.A. (2020). TREM-1 activation is a potential key regulator in driving severe pathogenesis of enterovirus A71 infection. Sci. Rep..

[43] Campbell G.R., To R.K., Spector S.A. (2019). TREM-1 Protects HIV-1-Infected Macrophages from Apoptosis through Maintenance of Mitochondrial Function. mBio.

[44] Hyun J., McMahon R.S., Lang A.L., Edwards J.S., Badilla A.D., Greene M.E., Stone G.W., Pallikkuth S., Stevenson M., Dykxhoorn D.M. (2019). HIV and HCV augments inflammatory responses through increased TREM-1 expression and signaling in Kupffer and Myeloid cells. PLoS Pathog..

[45] Kumar M., Belcaid M., Nerurkar V.R. (2016). Identification of host genes leading to West Nile virus encephalitis in mice brain using RNA-seq analysis. Sci. Rep..

[46] Mohamadzadeh M., Coberley S.S., Olinger G.G., Kalina W.V., Ruthel G., Fuller C.L., Swenson D.L., Pratt W.D., Kuhns D.B., Schmaljohn A.L. (2006). Activation of triggering receptor expressed on myeloid cells-1 on human neutrophils by marburg and ebola viruses. J. Virol..

[47] Mukhamedova N., Hoang A., Dragoljevic D., Dubrovsky L., Pushkarsky T., Low H., Ditiatkovski M., Fu Y., Ohkawa R., Meikle P.J. (2019). Exosomes containing HIV protein Nef reorganize lipid rafts potentiating inflammatory response in bystander cells. PLoS Pathog..

[48] Yuan Z., Fan X., Staitieh B., Bedi C., Spearman P., Guidot D.M., Sadikot R.T. (2017). HIV-related proteins prolong macrophage survival through induction of Triggering receptor expressed on myeloid cells-1. Sci. Rep..

[49] Bosco M.C., Pierobon D., Blengio F., Raggi F., Vanni C., Gattorno M., Eva A., Novelli F., Cappello P., Giovarelli M. (2011). Hypoxia modulates the gene expression profile of immunoregulatory receptors in human mature dendritic cells: Identification of TREM-1 as a novel hypoxic marker in vitro and in vivo. Blood.

[50] Bouchon A., Dietrich J., Colonna M. (2000). Cutting edge: Inflammatory responses can be triggered by TREM-1, a novel receptor expressed on neutrophils and monocytes. J. Immunol..

[51] Chen L.C., Laskin J.D., Gordon M.K., Laskin D.L. (2008). Regulation of TREM expression in hepatic macrophages and endothelial cells during acute endotoxemia. Exp. Mol. Pathol..

[52] Gingras M.C., Lapillonne H., Margolin J.F. (2002). TREM-1, MDL-1, and DAP12 expression is associated with a mature stage of myeloid development. Mol. Immunol..

[53] Rigo I., McMahon L., Dhawan P., Christakos S., Yim S., Ryan L.K., Diamond G. (2012). Induction of triggering receptor expressed on myeloid cells (TREM-1) in airway epithelial cells by 1,25(OH)_2_ vitamin D_3_. Innate Immun..

[54] Schmausser B., Endrich S., Beier D., Moran A.P., Burek C.J., Rosenwald A., Rieckmann P., Müller-Hermelink H.K., Eck M. (2008). Triggering receptor expressed on myeloid cells-1 (TREM-1) expression on gastric epithelium: Implication for a role of TREM-1 in Helicobacter pylori infection. Clin. Exp. Immunol..

[55] Tammaro A., Derive M., Gibot S., Leemans J.C., Florquin S., Dessing M.C. (2017). TREM-1 and its potential ligands in non-infectious diseases: From biology to clinical perspectives. Pharmacol. Ther..

[56] Bleharski J.R., Kiessler V., Buonsanti C., Sieling P.A., Stenger S., Colonna M., Modlin R.L. (2003). A role for triggering receptor expressed on myeloid cells-1 in host defense during the early-induced and adaptive phases of the immune response. J. Immunol..

[57] Boufenzer A., Carrasco K., Jolly L., Brustolin B., Di-Pillo E., Derive M., Gibot S. (2021). Potentiation of NETs release is novel characteristic of TREM-1 activation and the pharmacological inhibition of TREM-1 could prevent from the deleterious consequences of NETs release in sepsis. Cell. Mol. Immunol..

[58] Dower K., Ellis D.K., Saraf K., Jelinsky S.A., Lin L.L. (2008). Innate immune responses to TREM-1 activation: Overlap, divergence, and positive and negative cross-talk with bacterial lipopolysaccharide. J. Immunol..

[59] Netea M.G., Azam T., Ferwerda G., Girardin S.E., Kim S.H., Dinarello C.A. (2006). Triggering receptor expressed on myeloid cells-1 (TREM-1) amplifies the signals induced by the NACHT-LRR (NLR) pattern recognition receptors. J. Leukoc. Biol..

[60] Prüfer S., Weber M., Sasca D., Teschner D., Wölfel C., Stein P., Stassen M., Schild H., Radsak M.P. (2014). Distinct signaling cascades of TREM-1, TLR and NLR in neutrophils and monocytic cells. J. Innate Immun..

[61] Radsak M.P., Salih H.R., Rammensee H.G., Schild H. (2004). Triggering receptor expressed on myeloid cells-1 in neutrophil inflammatory responses: Differential regulation of activation and survival. J. Immunol..

[62] Schenk M., Bouchon A., Seibold F., Mueller C. (2007). TREM-1--expressing intestinal macrophages crucially amplify chronic inflammation in experimental colitis and inflammatory bowel diseases. J. Clin. Investig..

[63] Denning N.L., Aziz M., Murao A., Gurien S.D., Ochani M., Prince J.M., Wang P. (2020). Extracellular CIRP as an endogenous TREM-1 ligand to fuel inflammation in sepsis. JCI Insight.

[64] Fu L., Han L., Xie C., Li W., Lin L., Pan S., Zhou Y., Li Z., Jin M., Zhang A. (2017). Identification of Extracellular Actin As a Ligand for Triggering Receptor Expressed on Myeloid Cells-1 Signaling. Front. Immunol..

[65] Read C.B., Kuijper J.L., Hjorth S.A., Heipel M.D., Tang X., Fleetwood A.J., Dantzler J.L., Grell S.N., Kastrup J., Wang C. (2015). Cutting Edge: Identification of neutrophil PGLYRP1 as a ligand for TREM-1. J. Immunol..

[66] Wu J., Li J., Salcedo R., Mivechi N.F., Trinchieri G., Horuzsko A. (2012). The proinflammatory myeloid cell receptor TREM-1 controls Kupffer cell activation and development of hepatocellular carcinoma. Cancer Res..

[67] Tang D., Chen M., Huang X., Zhang G., Zeng L., Zhang G., Wu S., Wang Y. (2023). SRplot: A free online platform for data visualization and graphing. PLoS ONE.

[68] Lei C., Yang J., Hu J., Sun X. (2021). On the Calculation of TCID(50) for Quantitation of Virus Infectivity. Virol. Sin..

[69] Davis B.D., Dulbecco R., Eisen H.N., Ginsberg H.S., Wood W.B. (1972). Nature of viruses. Microbiology.

[70] Berman H.M., Westbrook J., Feng Z., Gilliland G., Bhat T.N., Weissig H., Shindyalov I.N., Bourne P.E. (2000). The Protein Data Bank. Nucleic Acids Res..

[71] Ge Y., Mansell A., Ussher J.E., Brooks A.E., Manning K., Wang C.J., Taylor J.A. (2013). Rotavirus NSP4 Triggers Secretion of Proinflammatory Cytokines from Macrophages via Toll-Like Receptor 2. J. Virol..

[72] Graham K.L., Fleming F.E., Halasz P., Hewish M.J., Nagesha H.S., Holmes I.H., Takada Y., Coulson B.S. (2005). Rotaviruses interact with alpha4beta7 and alpha4beta1 integrins by binding the same integrin domains as natural ligands. J. Gen. Virol..

[73] Mirdita M., Schütze K., Moriwaki Y., Heo L., Ovchinnikov S., Steinegger M. (2022). ColabFold: Making protein folding accessible to all. Nat. Methods.

[74] Williams C.J., Headd J.J., Moriarty N.W., Prisant M.G., Videau L.L., Deis L.N., Verma V., Keedy D.A., Hintze B.J., Chen V.B. (2018). MolProbity: More and better reference data for improved all-atom structure validation. Protein Sci. A Publ. Protein Soc..

[75] Jones G., Jindal A., Ghani U., Kotelnikov S., Egbert M., Hashemi N., Vajda S., Padhorny D., Kozakov D. (2022). Elucidation of protein function using computational docking and hotspot analysis by ClusPro and FTMap. Biol. Crystallogr..

[76] Kozakov D., Beglov D., Bohnuud T., Mottarella S.E., Xia B., Hall D.R., Vajda S. (2013). How good is automated protein docking?. Proteins.

[77] Kozakov D., Hall D.R., Xia B., Porter K.A., Padhorny D., Yueh C., Beglov D., Vajda S. (2017). The ClusPro web server for protein-protein docking. Nat. Protoc..

[78] Abraham M.J., Murtola T., Schulz R., Páll S., Smith J.C., Hess B., Lindahl E. (2015). GROMACS: High performance molecular simulations through multi-level parallelism from laptops to supercomputers. SoftwareX.

[79] Sukhwal A., Sowdhamini R. (2013). Oligomerisation status and evolutionary conservation of interfaces of protein structural domain superfamilies. Mol. Biosyst..

[80] Laskowski R.A. (2022). PDBsum1: A standalone program for generating PDBsum analyses. Protein Sci..

[81] Laskowski R.A., Swindells M.B. (2011). LigPlot+: Multiple ligand-protein interaction diagrams for drug discovery. J. Chem. Inf. Model..

[82] Weng G., Wang E., Wang Z., Liu H., Zhu F., Li D., Hou T. (2019). HawkDock: A web server to predict and analyze the protein-protein complex based on computational docking and MM/GBSA. Nucleic Acids Res..

[83] Xue L.C., Rodrigues J.P., Kastritis P.L., Bonvin A.M., Vangone A. (2016). PRODIGY: A web server for predicting the binding affinity of protein-protein complexes. Bioinformatics.

[84] Moal I.H., Jiménez-García B., Fernández-Recio J. (2015). CCharPPI web server: Computational characterization of protein-protein interactions from structure. Bioinformatics.

[85] Roy A.A., Dhawanjewar A.S., Sharma P., Singh G., Madhusudhan M.S. (2019). Protein Interaction Z Score Assessment (PIZSA): An empirical scoring scheme for evaluation of protein-protein interactions. Nucleic Acids Res..

[86] Bessho K., Shanmukhappa K., Sheridan R., Shivakumar P., Mourya R., Walters S., Kaimal V., Dilbone E., Jegga A.G., Bezerra J.A. (2013). Integrative genomics identifies candidate microRNAs for pathogenesis of experimental biliary atresia. BMC Syst. Biol..

[87] Tsuge M., Oka T., Yamashita N., Saito Y., Fujii Y., Nagaoka Y., Yashiro M., Tsukahara H., Morishima T. (2014). Gene expression analysis in children with complex seizures due to influenza A(H1N1)pdm09 or rotavirus gastroenteritis. J. Neurovirol..

[88] DeBerg H.A., Zaidi M.B., Altman M.C., Khaenam P., Gersuk V.H., Campos F.D., Perez-Martinez I., Meza-Segura M., Chaussabel D., Banchereau J. (2018). Shared and organism-specific host responses to childhood diarrheal diseases revealed by whole blood transcript profiling. PLoS ONE.

[89] Kelker M.S., Foss T.R., Peti W., Teyton L., Kelly J.W., Wüthrich K., Wilson I.A. (2004). Crystal Structure of Human Triggering Receptor Expressed on Myeloid Cells 1 (TREM-1) at 1.47Å. J. Mol. Biol..

[90] de Oliveira Matos A., Dos Santos Dantas P.H., Colmenares M.T.C., Sartori G.R., Silva-Sales M., Da Silva J.H.M., Neves B.J., Andrade C.H., Sales-Campos H. (2023). The CDR3 region as the major driver of TREM-1 interaction with its ligands, an in silico characterization. Comput. Struct. Biotechnol. J..

[91] Jin M.S., Kim S.E., Heo J.Y., Lee M.E., Kim H.M., Paik S.G., Lee H., Lee J.O. (2007). Crystal structure of the TLR1-TLR2 heterodimer induced by binding of a tri-acylated lipopeptide. Cell.

[92] Fortin C.F., Lesur O., Fulop T. (2007). Effects of TREM-1 activation in human neutrophils: Activation of signaling pathways, recruitment into lipid rafts and association with TLR4. Int. Immunol..

[93] Murao A., Arif A., Brenner M., Denning N.L., Jin H., Takizawa S., Nicastro B., Wang P., Aziz M. (2020). Extracellular CIRP and TREM-1 axis promotes ICAM-1-Rho-mediated NETosis in sepsis. FASEB J..

[94] Chen N., Jin J., Qiao B., Gao Z., Tian Y., Ping J. (2025). JNK kinase promotes inflammatory responses by inducing the expression of the inflammatory amplifier TREM1 during influenza a virus infection. Virus Res..

[95] Weber B., Schuster S., Zysset D., Rihs S., Dickgreber N., Schürch C., Riether C., Siegrist M., Schneider C., Pawelski H. (2014). TREM-1 deficiency can attenuate disease severity without affecting pathogen clearance. PLoS Pathog..

[96] Wu X., Zhao W., Miao Q., Shi S., Wei B., Luo L., Cai B. (2024). CCR2+TREM-1+ monocytes promote natural killer T cell dysfunction contributing towards HBV disease progression. Immunol. Res..

[97] Wu X., Cai B., Lu W., Fu Y., Wei B., Niu Q., Su Z., Li Y., Wang L. (2021). HBV upregulated triggering receptor expressed on myeloid cells-1 (TREM-1) expression on monocytes participated in disease progression through NF-Kb pathway. Clin. Immunol..

[98] Alipanah-Lechner N., Hurst-Hopf J., Delucchi K., Swigart L., Willmore A., LaCombe B., Dewar R., Lane H.C., Lallemand P., Liu K.D. (2024). Novel subtypes of severe COVID-19 respiratory failure based on biological heterogeneity: A secondary analysis of a randomized controlled trial. Crit. Care.

[99] de Nooijer A.H., Grondman I., Lambden S., Kooistra E.J., Janssen N.A.F., Kox M., Pickkers P., Joosten L.A.B., van de Veerdonk F.L., Derive M. (2021). Increased sTREM-1 plasma concentrations are associated with poor clinical outcomes in patients with COVID-19. Biosci. Rep..

[100] Fan R., Cheng Z., Huang Z., Yang Y., Sun N., Hu B., Hou P., Liu B., Huang C., Liu S. (2023). TREM-1, TREM-2 and their association with disease severity in patients with COVID-19. Ann. Med..

[101] Paranga T.G., Pavel-Tanasa M., Constantinescu D., Plesca C.E., Petrovici C., Miftode I.L., Moscalu M., Cianga P., Miftode E.G. (2023). Comparison of C-reactive protein with distinct hyperinflammatory biomarkers in association with COVID-19 severity, mortality and SARS-CoV-2 variants. Front. Immunol..

[102] Colmenares M.T.C., Matos A.O., Dantas P., Neto J., Neves B.J., Gardinassi L.G.A., Silva-Sales M., Sales-Campos H. (2025). TREM-1 as a Potential Coreceptor in Norovirus Pathogenesis: Insights from Transcriptomic Analysis and Molecular Docking. ACS Omega.

[103] Vinolo E., Maillefer M., Jolly L., Colné N., Meiffren G., Carrasco K., Derive M. (2024). The potential of targeting TREM-1 in IBD. Adv. Pharmacol..

[104] de Oliveira Matos A., Henrique Dos Santos Dantas P., Rodrigues do Carmo Neto J., Contreras Colmenares M.T., Felice A.G., de Castro Soares S., Silva-Sales M., Sales-Campos H. (2025). Uncovering the role of TREM-1 in celiac disease: In silico insights into the recognition of gluten-derived peptides and inflammatory mechanisms. Comput. Biol. Med..

[105] Altay F.A., Elaldi N., Şentürk G., Altin N., Gözel M.G., Albayrak Y., Şencan İ. (2016). Serum sTREM-1 level is quite higher in Crimean Congo Hemorrhagic Fever, a viral infection. J. Med. Virol..

[106] Ruiz-Pacheco J.A., Vivanco-Cid H., Izaguirre-Hernández I.Y., Estrada-García I., Arriaga-Pizano L., Chacón-Salinas R., Fonseca-Coronado S., Vaughan G., Tovar K.R., Rivera-Osorio M.P. (2014). TREM-1 modulation during early stages of dengue virus infection. Immunol. Lett..

[107] Carrasco K., Boufenzer A., Jolly L., Le Cordier H., Wang G., Heck A.J., Cerwenka A., Vinolo E., Nazabal A., Kriznik A. (2019). TREM-1 multimerization is essential for its activation on monocytes and neutrophils. Cell. Mol. Immunol..

[108] Gómez-Piña V., Soares-Schanoski A., Rodríguez-Rojas A., Del Fresno C., García F., Vallejo-Cremades M.T., Fernández-Ruiz I., Arnalich F., Fuentes-Prior P., López-Collazo E. (2007). Metalloproteinases shed TREM-1 ectodomain from lipopolysaccharide-stimulated human monocytes. J. Immunol..

[109] Weiss G., Lai C., Fife M.E., Grabiec A.M., Tildy B., Snelgrove R.J., Xin G., Lloyd C.M., Hussell T. (2017). Reversal of TREM-1 ectodomain shedding and improved bacterial clearance by intranasal metalloproteinase inhibitors. Mucosal Immunol..

[110] Jolly L., Carrasco K., Salcedo-Magguilli M., Garaud J.J., Lambden S., van der Poll T., Mebazaa A., Laterre P.F., Gibot S., Boufenzer A. (2021). sTREM-1 is a specific biomarker of TREM-1 pathway activation. Cell. Mol. Immunol..

[111] Bruneel F., Tubach F., Mira J.P., Houze S., Gibot S., Huisse M.G., Megarbane B., Choquet C., Corne P., Peytel E. (2016). Imported falciparum malaria in adults: Host- and parasite-related factors associated with severity. The French prospective multicenter PALUREA cohort study. Intensive Care Med..

[112] Horst S.A., Linnér A., Beineke A., Lehne S., Höltje C., Hecht A., Norrby-Teglund A., Medina E., Goldmann O. (2013). Prognostic value and therapeutic potential of TREM-1 in Streptococcus pyogenes- induced sepsis. J. Innate Immun..

[113] Kozik J.H., Trautmann T., Carambia A., Preti M., Lütgehetmann M., Krech T., Wiegard C., Heeren J., Herkel J. (2016). Attenuated viral hepatitis in Trem1-/- mice is associated with reduced inflammatory activity of neutrophils. Sci. Rep..

[114] Chen S.-M., Lin C.-P., Tsai J.-D., Chao Y.-H., Sheu J.-N. (2014). The Significance of Serum and Fecal Levels of Interleukin-6 and Interleukin-8 in Hospitalized Children with Acute Rotavirus and Norovirus Gastroenteritis. Pediatr. Neonatol..

[115] Di Fiore I.J.M., Holloway G., Coulson B.S. (2015). Innate immune responses to rotavirus infection in macrophages depend on MAVS but involve neither the NLRP3 inflammasome nor JNK and p38 signaling pathways. Virus Res..

[116] Gómez-Rial J., Curras-Tuala M.J., Rivero-Calle I., Rodríguez-Tenreiro C., Redondo-Collazo L., Gómez-Carballa A., Pardo-Seco J., Salas A., Martinón-Torres F. (2018). Rotavirus intestinal infection induces an oral mucosa cytokine response. PLoS ONE.

[117] Hagbom M., Hellysaz A., Istrate C., Nordgren J., Sharma S., de-Faria F.M., Magnusson K.E., Svensson L. (2021). The 5-HT(3) Receptor Affects Rotavirus-Induced Motility. J. Virol..

[118] Jiang B., Snipes-Magaldi L., Dennehy P., Keyserling H., Holman R.C., Bresee J., Gentsch J., Glass R.I. (2003). Cytokines as Mediators for or Effectors against Rotavirus Disease in Children. Clin. Vaccine Immunol..

[119] Kim J.-H., Kim K., Kim W. (2020). Genipin inhibits rotavirus-induced diarrhea by suppressing viral replication and regulating inflammatory responses. Sci. Rep..

[120] Deal E.M., Jaimes M.C., Crawford S.E., Estes M.K., Greenberg H.B. (2010). Rotavirus structural proteins and dsRNA are required for the human primary plasmacytoid dendritic cell IFNalpha response. PLoS Pathog..

[121] Hagbom M., Istrate C., Engblom D., Karlsson T., Rodriguez-Diaz J., Buesa J., Taylor J.A., Loitto V.M., Magnusson K.E., Ahlman H. (2011). Rotavirus stimulates release of serotonin (5-HT) from human enterochromaffin cells and activates brain structures involved in nausea and vomiting. PLoS Pathog..

[122] Hakim M.S., Ding S., Chen S., Yin Y., Su J., van der Woude C.J., Fuhler G.M., Peppelenbosch M.P., Pan Q., Wang W. (2018). TNF-α exerts potent anti-rotavirus effects via the activation of classical NF-κB pathway. Virus Res..

[123] Boraschi D. (2022). What Is IL-1 for? The Functions of Interleukin-1 Across Evolution. Front. Immunol..

[124] Nishimoto N., Kishimoto T. (2006). Interleukin 6: From bench to bedside. Nat. Clin. Pract. Rheumatol..

[125] Aggeletopoulou I., Kalafateli M., Tsounis E.P., Triantos C. (2024). Exploring the role of IL-1β in inflammatory bowel disease pathogenesis. Front. Med..

[126] Alhendi A., Naser S.A. (2023). The dual role of interleukin-6 in Crohn’s disease pathophysiology. Front. Immunol..

[127] Kandil H.M., Berschneider H.M., Argenzio R.A. (1994). Tumour necrosis factor alpha changes porcine intestinal ion transport through a paracrine mechanism involving prostaglandins. Gut.

[128] Oprins J.C., Meijer H.P., Groot J.A. (2000). TNF-alpha potentiates the ion secretion induced by muscarinic receptor activation in HT29cl.19A cells. Am. J. Physiol. Cell Physiol..

[129] Roth J., De Souza G.E. (2001). Fever induction pathways: Evidence from responses to systemic or local cytokine formation. Braz. J. Med. Biol. Res..

[130] Mota C.M.D., Madden C.J. (2022). Neural circuits mediating circulating interleukin-1β-evoked fever in the absence of prostaglandin E2 production. Brain Behav. Immun..

[131] Lopez S., Arias C.F. (2006). Early steps in rotavirus cell entry. Curr. Top. Microbiol. Immunol..

[132] Venkataram Prasad B.V., Shanker S., Hu L., Choi J.M., Crawford S.E., Ramani S., Czako R., Atmar R.L., Estes M.K. (2014). Structural basis of glycan interaction in gastroenteric viral pathogens. Curr. Opin. Virol..

[133] Gutiérrez M., Isa P., Sánchez-San Martin C., Pérez-Vargas J., Espinosa R., Arias C.F., López S. (2010). Different rotavirus strains enter MA104 cells through different endocytic pathways: The role of clathrin-mediated endocytosis. J. Virol..

[134] Arias C.F., Silva-Ayala D., López S. (2015). Rotavirus entry: A deep journey into the cell with several exits. J. Virol..

[135] Cuadras M.A., Bordier B.B., Zambrano J.L., Ludert J.E., Greenberg H.B. (2006). Dissecting rotavirus particle-raft interaction with small interfering RNAs: Insights into rotavirus transit through the secretory pathway. J. Virol..

[136] Cuadras M.A., Greenberg H.B. (2003). Rotavirus infectious particles use lipid rafts during replication for transport to the cell surface in vitro and in vivo. Virology.

[137] Martínez M.A., López S., Arias C.F., Isa P. (2013). Gangliosides have a functional role during rotavirus cell entry. J. Virol..

[138] Simons K., Ehehalt R. (2002). Cholesterol, lipid rafts, and disease. J. Clin. Investig..

[139] Guerrero C.A., Méndez E., Zárate S., Isa P., López S., Arias C.F. (2000). Integrin alpha(v)beta(3) mediates rotavirus cell entry. Proc. Natl. Acad. Sci. USA.

[140] Sánchez-San Martín C., López T., Arias C.F., López S. (2004). Characterization of rotavirus cell entry. J. Virol..

[141] Ball J.M., Tian P., Zeng C.Q., Morris A.P., Estes M.K. (1996). Age-dependent diarrhea induced by a rotaviral nonstructural glycoprotein. Science.

[142] Begum N.A., Ishii K., Kurita-Taniguchi M., Tanabe M., Kobayashi M., Moriwaki Y., Matsumoto M., Fukumori Y., Azuma I., Toyoshima K. (2004). Mycobacterium bovis BCG cell wall-specific differentially expressed genes identified by differential display and cDNA subtraction in human macrophages. Infect. Immun..

[143] Zhang M., Zeng C.Q., Morris A.P., Estes M.K. (2000). A functional NSP4 enterotoxin peptide secreted from rotavirus-infected cells. J. Virol..

[144] Tsugawa T., Tatsumi M., Tsutsumi H. (2014). Virulence-associated genome mutations of murine rotavirus identified by alternating serial passages in mice and cell cultures. J. Virol..

