# Supplementary material for: TREM-1 Interacts with Rotavirus Proteins and Drives Inflammatory Responses: A Combined Experimental and Computational Approach
Source: Pathogens. 2025 Oct 10;14(10):1029. doi: 10.3390/pathogens14101029 (PMC12566830; doi:10.3390/pathogens14101029)
Supplement: Supplementary file 1 [file pathogens-14-01029-s001.zip › pathogens-3911255-supplementary.pdf]

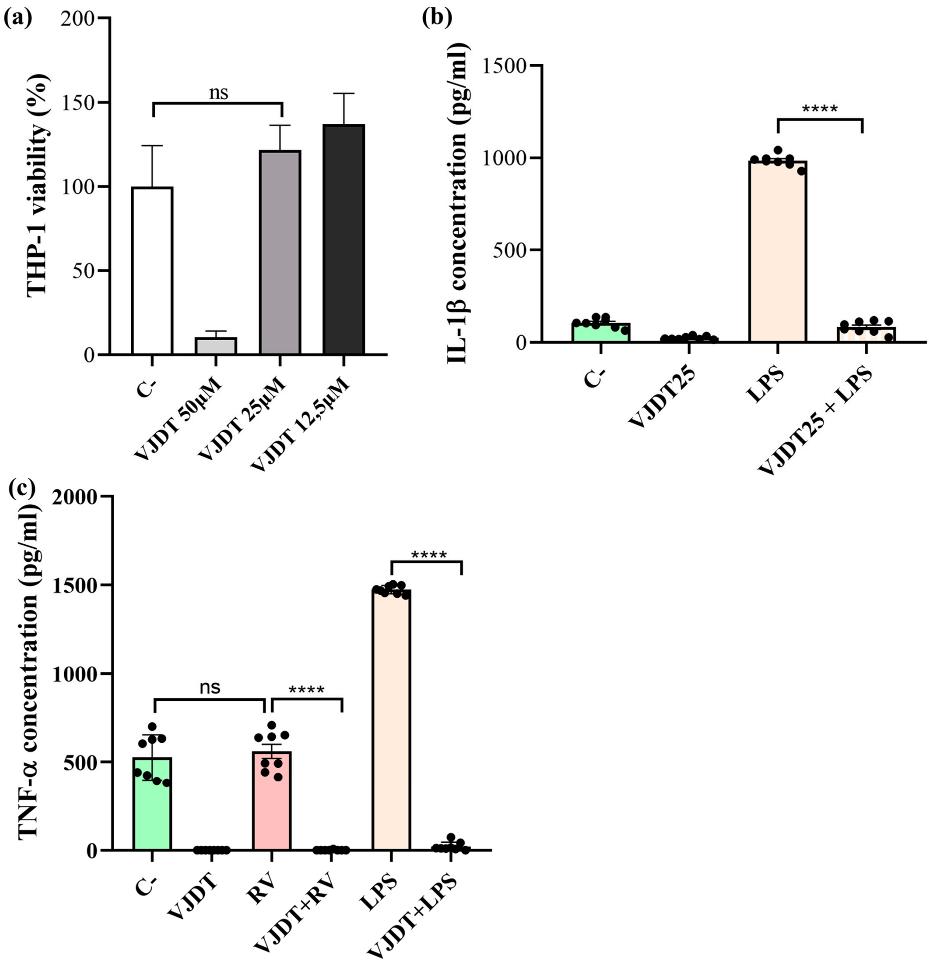

Figure S1. TREM-1 inhibition impact in cytokine production. (**a**) Toxicity evaluation of different VJDT concentrations in THP-1. Cells (5.10[4] cells/well) were treated for 24 hours with 12,5 µM, 25 µM and 50µM of VJDT, and the cell viability was assessed with the MTT assay. (**b**) IL-1β levels and (**c**) TNF-α levels, assessed in the cell culture supernatant of THP-1 cells, with or without pre-treatment for 1 hour with VJDT at 25µM, followed by LPS or RV exposure, for 24 hours. Concentrations (pg/ml) were assessed by ELISA. C-: negative control, ie., cells in the presence of the cell media alone. For both MTT and ELISA assays, statistical analysis were conducted by one-way ANOVA followed by Turkey's test. The column bar graphs depict mean values with the standard error of the mean (SEM), ****p<0.0001, ns: non-significant.

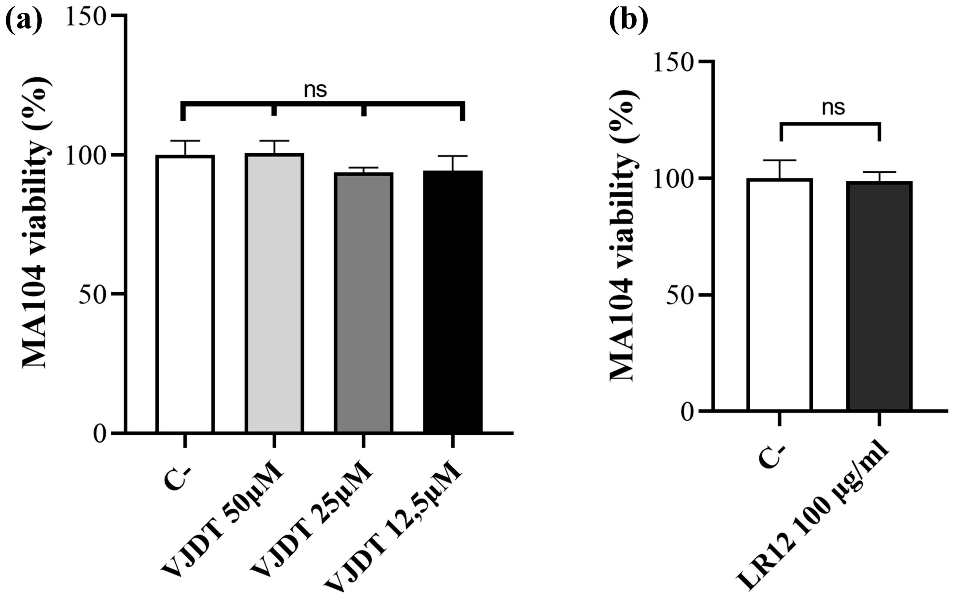

Figure S2. Evaluation of the toxicity of TREM-1 inhibitors in MA104 cells. (**a** and **b**) Toxicity evaluation of VJDT (in different concentrations) and of LR12 (100 μg/ml) in MA104. Cells (5.10^4 cells/well) were treated for 24 hours with the inhibitors, and the cell viability was assessed with the MTT assay. C-: negative control, ie., cells in the presence of the cell media alone. Statistical analyses were conducted by one-way ANOVA followed by Turkey's test. The column bar graphs depict mean values with the standard error of the mean (SEM), ns: non-significant.

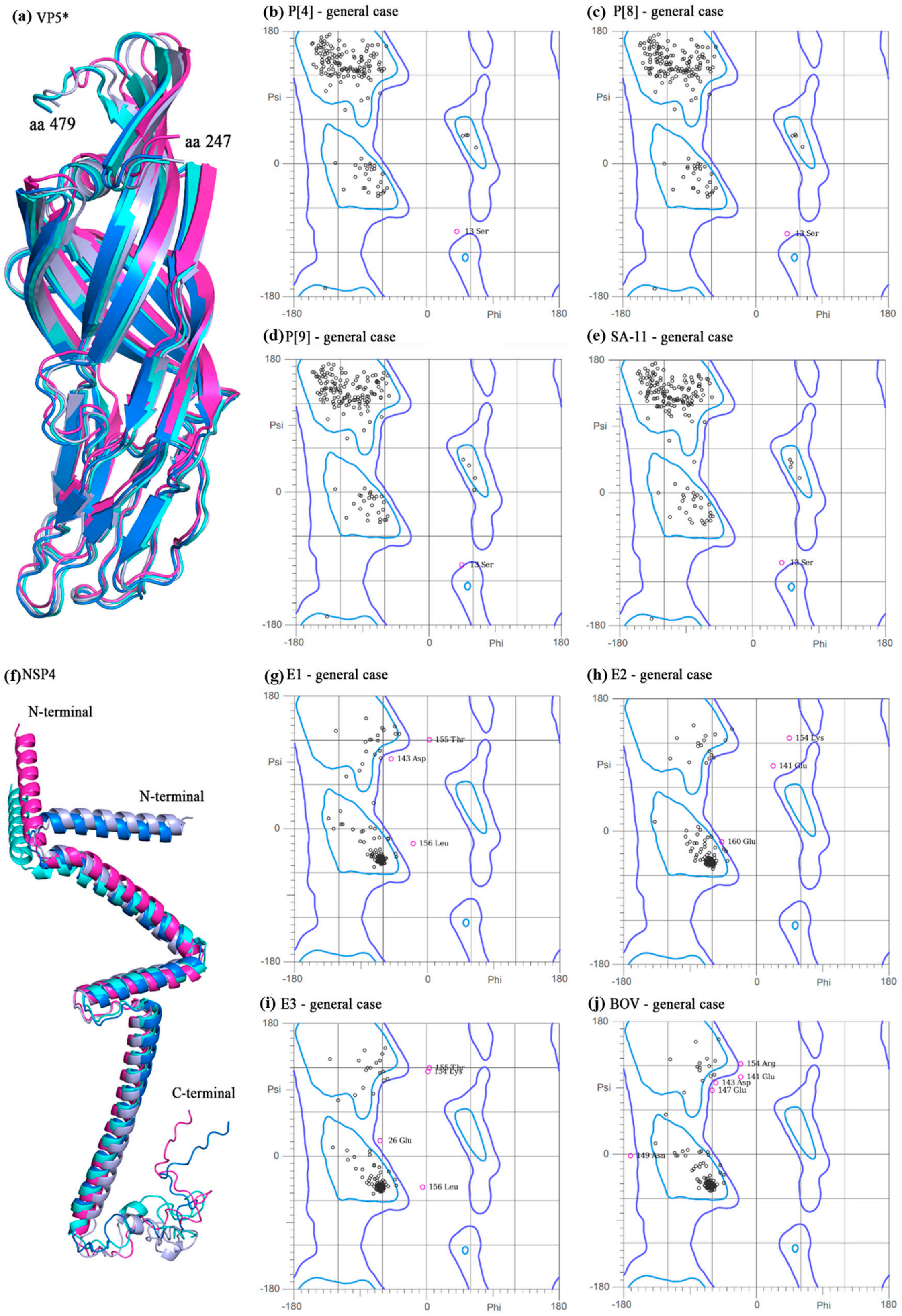

Figure S3. Structure prediction of VP5* and NSP4 proteins. (**a**) Alignment of the predicted P[4], P[8], P[9] and SA-11 VP5* proteins. Ramachandran plots for P[4] (**b**), P[8] (**c**), P[9] (**d**) and SA-11 (**e**) VP5* proteins. (**f**) Alignment of the predicted E1, E3, E3 and bovine rotavirus proteins. Ramachandran plots for E1 (**g**), E2 (**h**), E3 (**i**) and bovine rotavirus (**j**) NSP4 proteins.

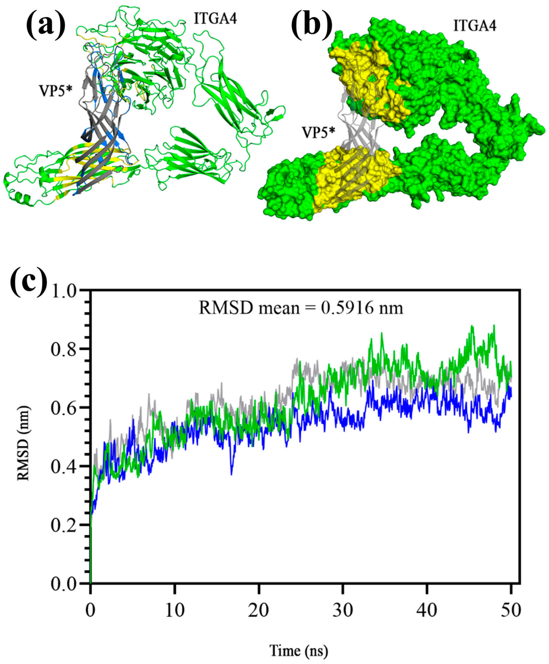

Figure S4. Interaction between SA-11 VP5* and integrin alpha 4 (ITGA4). (**a** and **b**) Predicted VP5*/ITGA4 complex. ITGA4 in green with interacting residues in yellow, SA-11 VP5* in gray with interacting residues in blue. (**c**) Graph displaying the root mean square deviation (RMSD) values throughout the 50 nanoseconds (ns) molecular dynamics simulations between ITGA4 and SA-11 VP5* protein. Each blue, green and gray line represents a replicate.

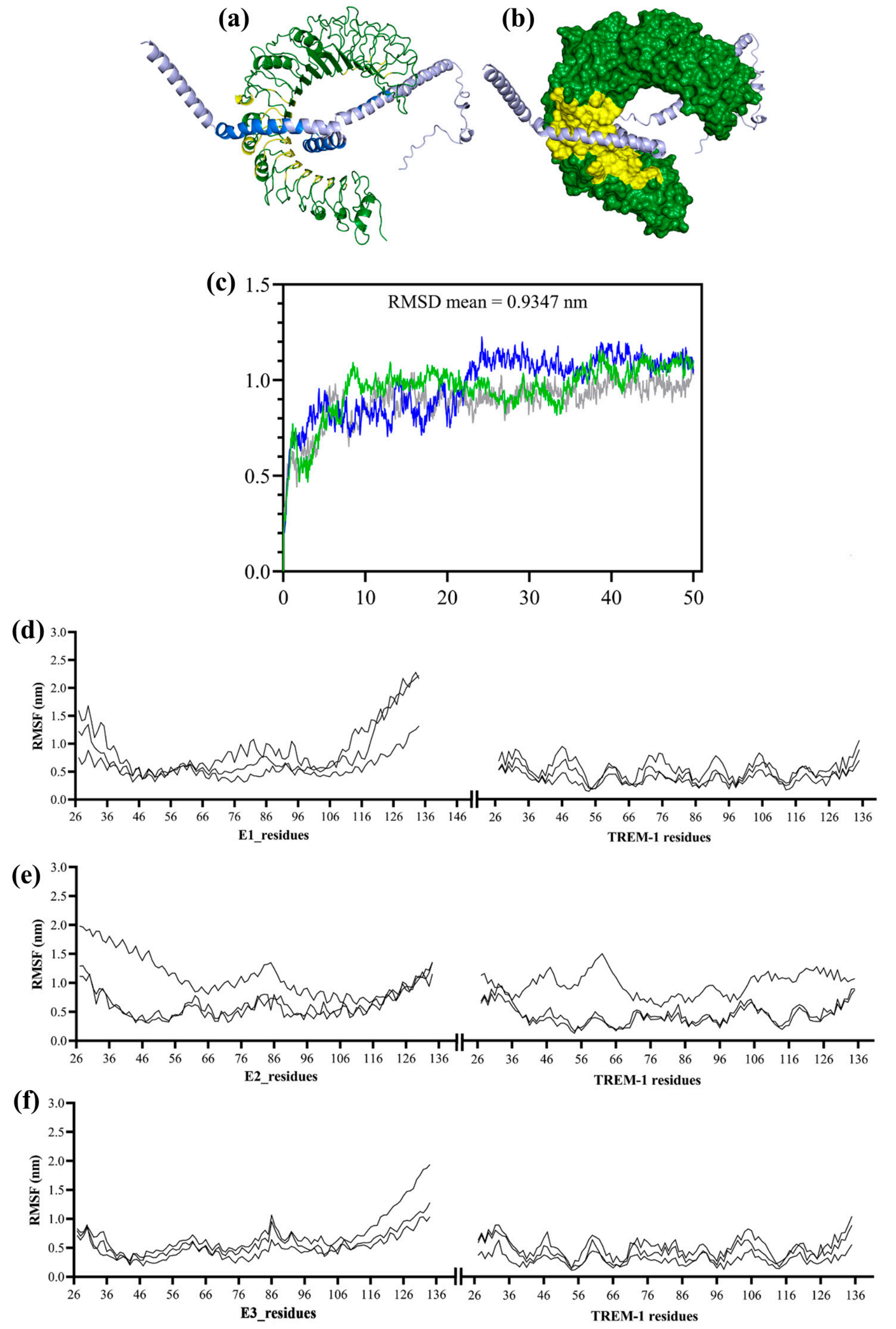

Figure S5. Interaction between bovine NSP4 and toll-like receptor 2 (TLR2). (**a** and **b**) Predicted NSP4/TLR2 complex. TLR2 in green with interacting residues in yellow, bovine NSP4 in lilac with interacting residues in blue. (**c**) Graph displaying the root mean square deviation (RMSD) values throughout the 50 nanoseconds (ns) molecular dynamics simulations between TLR2 and bovine NSP4 protein. Each blue, green and gray line represents a replicate. (**d** to **f**) Graphs displaying the root mean square fluctuation (RMSF) values for the TREM-1/E1 (**d**), TREM-1/E2 (**e**) and TREM-1/E3 (**f**) complexes. Each line represents a replicate.
